# Status and potential of bacterial genomics for public health practice: a scoping review

**DOI:** 10.1186/s13012-019-0930-2

**Published:** 2019-08-13

**Authors:** Nina Van Goethem, Tine Descamps, Brecht Devleesschauwer, Nancy H. C. Roosens, Nele A. M. Boon, Herman Van Oyen, Annie Robert

**Affiliations:** 1 Department of Epidemiology and public health, Sciensano, J. Wytsmanstraat 14, 1050 Brussels, Belgium; 2 Department of Epidemiology and Biostatistics, Institut de recherche expérimentale et clinique, Faculty of Public Health, Université catholique de Louvain, Clos Chapelle-aux-champs 30, 1200 Woluwe-Saint-Lambert, Belgium; 3 Department of Veterinary Public Health and Food Safety, Faculty of Veterinary Medicine, Ghent University, Salisburylaan 133, 9820 Merelbeke, Belgium; 4 Transversal Activities in Applied Genomics, Sciensano, J. Wytsmanstraat 14, 1050 Brussels, Belgium; 5 Department of Public Health and Primary Care, Faculty of Medicine, Ghent University, De Pintelaan 185, 9000 Ghent, Belgium

**Keywords:** Public health practice, Bacterial infections, Next-generation sequencing, Whole-genome sequencing, Genomics, Epidemiology, Scoping review

## Abstract

**Background:**

Next-generation sequencing (NGS) is increasingly being translated into routine public health practice, affecting the surveillance and control of many pathogens. The purpose of this scoping review is to identify and characterize the recent literature concerning the application of bacterial pathogen genomics for public health practice and to assess the added value, challenges, and needs related to its implementation from an epidemiologist’s perspective.

**Methods:**

In this scoping review, a systematic PubMed search with forward and backward snowballing was performed to identify manuscripts in English published between January 2015 and September 2018. Included studies had to describe the application of NGS on bacterial isolates within a public health setting. The studied pathogen, year of publication, country, number of isolates, sampling fraction, setting, public health application, study aim, level of implementation, time orientation of the NGS analyses, and key findings were extracted from each study. Due to a large heterogeneity of settings, applications, pathogens, and study measurements, a descriptive narrative synthesis of the eligible studies was performed.

**Results:**

Out of the 275 included articles, 164 were outbreak investigations, 70 focused on strategy-oriented surveillance, and 41 on control-oriented surveillance. Main applications included the use of whole-genome sequencing (WGS) data for (1) source tracing, (2) early outbreak detection, (3) unraveling transmission dynamics, (4) monitoring drug resistance, (5) detecting cross-border transmission events, (6) identifying the emergence of strains with enhanced virulence or zoonotic potential, and (7) assessing the impact of prevention and control programs. The superior resolution over conventional typing methods to infer transmission routes was reported as an added value, as well as the ability to simultaneously characterize the resistome and virulome of the studied pathogen. However, the full potential of pathogen genomics can only be reached through its integration with high-quality contextual data.

**Conclusions:**

For several pathogens, it is time for a shift from proof-of-concept studies to routine use of WGS during outbreak investigations and surveillance activities. However, some implementation challenges from the epidemiologist’s perspective remain, such as data integration, quality of contextual data, sampling strategies, and meaningful interpretations. Interdisciplinary, inter-sectoral, and international collaborations are key for an appropriate genomics-informed surveillance.

**Electronic supplementary material:**

The online version of this article (10.1186/s13012-019-0930-2) contains supplementary material, which is available to authorized users.

## Introduction

The advent and continuous improvement of sequencing technologies, especially the shift to next-generation sequencing (NGS), provides many opportunities for the management of infectious diseases. Sequence information can identify a pathogen and its specific characteristics, as well as its relatedness to other pathogens. Compared to Sanger sequencing, NGS technologies allow a faster and cheaper way to sequence large amounts of nucleotides. As such, NGS can be viewed as a tool that makes whole-genome sequencing (WGS) accessible [1]. In contrast to genotyping, where only small parts of the genome are assessed, WGS provides characteristics of the entire genome of the infectious isolates, thereby combining maximal strain discrimination and the ability to link the genotype with clinically and epidemiologically relevant phenotypes [2, 3]. Sequence variations, such as single-nucleotide polymorphisms (SNPs), insertions/deletions, and accessory genes can be identified following bioinformatics analyses [1]. Highly discriminatory subtyping following WGS is accomplished based on either SNPs or allelic variation [5]. With decreasing costs and increasing laboratory and bioinformatics capacities, we are currently transitioning to genomic epidemiology, as whole pathogen genomes are available at the level of the population [3]. Adding genomic data to epidemiological analyses of infectious diseases greatly benefits disease prevention and control [1, 2]. During the last decade, NGS is no longer limited to research settings and is being rapidly translated into public health practice [4, 5].

**Fig. 1:**
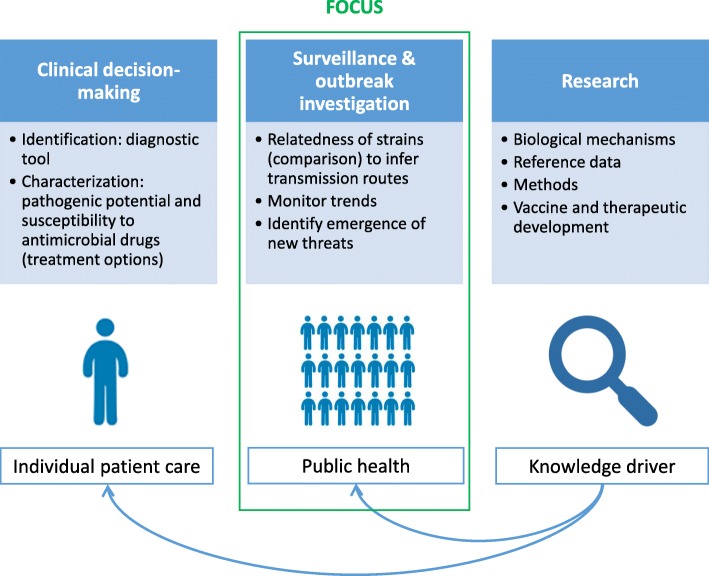
Focus of the scoping review on pathogen genomics for public health practice. Different domains in the field of infectious diseases require access to the same pathogen genomic data. Whole-genome sequencing (WGS) has the ability to inform and improve individual patient care, by identifying the species, determining its pathogenic potential, and testing its susceptibility to antimicrobial drugs. WGS also provides data for public health surveillance about the relatedness of the pathogen to other strains to investigate transmission routes, monitor trends over time, and allow the identification and control of outbreaks and new threats. Research is a knowledge driver providing reference data, methods, and a deeper understanding about the underlying biological mechanisms to the other domains. The focus of this scoping review is on the use of WGS as a public health tool, i.e., at the level of the population

This review focuses on the applications of NGS to the population-level management of bacterial infections (Fig. 1). This includes the use of WGS to study the relatedness of isolates in order to understand transmission dynamics, to detect and control outbreaks, to monitor trends, and to identify the emergence of new threats. More specifically, this review discusses the applications of pathogen genomics that lead to actionable results from a public health point of view.

Public health activities related to infectious diseases can be classified as outbreak investigations, control-oriented surveillance, and strategy-oriented surveillance [3, 6]. The main objective of an outbreak investigation is to investigate the possible source(s) of infection and to implement effective and appropriate control measures to stop its further spread. Outbreak investigations are often hypothesis-driven and a reaction to a sudden increase in the number of cases [7]. In contrast, surveillance is the systematic collection, analysis, and dissemination of data for the planning, implementation, and evaluation of public health programs [8]. Baker et al. [6] differentiate between control-oriented and strategy-oriented surveillance, thereby providing a meaningful way to categorize the applications of molecular/genomic tools for disease surveillance [9]. This framework has also been adopted in the European Centre for Disease Prevention and Control (ECDC) roadmap for integration of molecular and genomic typing into European level surveillance and epidemic preparedness [10]. As defined by Baker et al. [6], the purpose of control-oriented surveillance is “to identify each occurrence of a particular disease, hazard, or other health-related event that requires a specific response, and to support the delivery of an effective intervention”. For example, control-oriented surveillance aims at the detection of outbreaks that require a specific response. Early outbreak detection can be achieved by prospectively genotyping as many consecutive cases in a population as possible to identify clusters of clonally linked isolates [3]. Baker et al. state that strategy-oriented surveillance aims “to provide information to support prevention strategies to reduce population risk” [6]. The aim is often to monitor long-term changes in epidemiology over larger geographic and population scales, requiring study designs that have a high degree of representativeness [9]. Strategy-oriented surveillance can for example detect the emergence of strains with enhanced virulence or drug resistance, help to identify risk factors associated with the transmission of specific strains, or predict the effectiveness of control programs such as vaccination campaigns [3].

Collective experience on the use of pathogen genomics for routine public health practice is spread across literature. WGS has been frequently used to aid outbreak investigations and routine surveillance at various levels (i.e., local, national, and international) and in different temporal scenarios (i.e., retrospective and prospective). The aim of this scoping review is to identify and characterize the recent literature concerning the application of NGS for public health practice, by (1) conducting a systematic search of the published literature, (2) mapping the characteristics of the identified studies, (3) describing the range of applications identified, and (4) assessing the added value, challenges, and requirements related to its implementation. The purpose is to provide an epidemiologist’s perspective on the use of bacterial pathogen genomics in a public health context to complement previous reviews that focused on technical aspects, bioinformatics, diagnostics, or microbiology (i.e., the perspective of microbiologists, bioinformaticians, and clinicians) [2, 5, 11–16]. This review aims to summarize the experience gained and use it to further advance the implementation of pathogen genomics in routine public health.

## Methods

A scoping review methodology was chosen to provide an overview of the nature and extent of the literature on this topic via systematically searching, selecting, and summarizing evidence, rather than a traditional systematic review that often focuses on specific outcomes [17]. This review was conducted according to the Preferred Reporting Items for Systematic Reviews and Meta-Analyses (PRISMA) guidelines [see Additional file 1] [18], adapted for use in a scoping review as appropriate, and adhering to the methodology outlined in The Joanna Briggs Institute Manual for Scoping reviews [19]. In addition, the framework outlined by Arksey and O’Malley [20] in their methodological paper on scoping reviews was followed. A scoping review protocol was developed a priori to ensure reproducibility and transparency of the review methods [see Additional file 2].

### Eligibility criteria

The inclusion criteria were organized following the PCC (Population, Concept, and Context) elements. Studies had to include at least 2 individuals with a bacterial infection, and NGS had to be applied on the bacterial isolates. Consequently, non-human studies and case reports involving only one patient were excluded, as well as studies focusing on the host genome. Studies had to describe the application of NGS from a public health perspective (i.e., population-level). Therefore, the main study aims had to be within the context of an outbreak investigation, control-oriented surveillance, or strategy-oriented surveillance. Studies focusing on technical aspects, applying NGS solely for individual patient care, and using NGS primarily for research purposes were excluded. Further, only studies applying NGS within a real-life public health setting, as opposed to an experimental setting, and producing an output that can be directly translated into actionable results to benefit public health, were included. This also included proof-of-concept studies mimicking real-life public health situations. Studies published between January 2015 and September 2018 were included to consider the most current activities in this fast-evolving field. A full list of inclusion and exclusion criteria and a decision tree for study selection is provided in the additional material [see Additional files 3 and 4].

### Searching

The PubMed search engine was used to identify manuscripts in English published between 1/1/2015 and 4/9/2018. In addition, reference lists of included studies and other reviews were examined (i.e., backward snowballing). Also, forward snowballing was performed by identifying relevant documents that cited the included studies, using the Google Scholar search engine.

Three domains were included in the search using the PubMed search engine: “bacterial infections,” “next generation sequencing,” and “public health.” Each domain had several search terms. Free text search and MeSH term search were combined. The search was pre-tested to determine the most effective balance of sensitivity and specificity in the identification of potentially relevant citations. The ability of the electronic search to capture all relevant primary research was verified by hand-searching reference lists from other reviews on the topic. The final search string is reported in the additional material [see Additional file 5]. The initial search was conducted on March 24, 2018, and was updated on September 4, 2018, selecting the date range “March 1, 2018, to September 4, 2018.”

### Screening

A first screening phase based on titles and abstracts was conducted [NVG], and out-of-topic studies were excluded. A second screening stage based on the full texts was conducted in duplicate by two independent reviewers [NVG, TD] using a standardized eligibility form. If no consensus could be reached between the two reviewers, a third reviewer [NB] helped to resolve the disagreement.

### Data extraction

Data extraction was performed by [NVG] using an extraction form that was designed for the purpose of this review through an iterative process [see Additional file 6]. Information regarding the studied pathogen, country, year of publication, number of isolates, sampling fraction and time orientation of the NGS analyses, setting, public health application, study aim(s), and level of implementation was extracted from each included study. In addition, key findings related to the use of NGS were summarized for every study.

### Data synthesis

The main characteristics were summarized in tabular form (as per data extraction pro-forma as well as a numerical summary), with an accompanying narrative summary, based on the key findings extracted from every study, describing how the results relate to the review objective and question. The studies were categorized based on the public health application and study aim in order to structure the narrative summary. For the study aim, multiple classifications per article were allowed.

## Results

### Search results

**Fig. 2:**
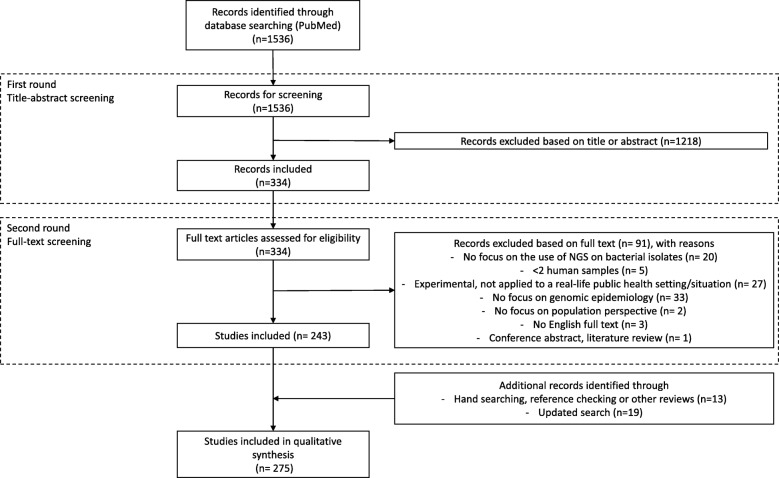
Preferred Reporting Items for Systematic Reviews and Meta-Analyses (PRISMA) flow diagram

The study selection process is summarized as a PRISMA flow diagram in Fig. 2. A total of 1549 studies were identified through the initial database search, hand searching, reference checking, and other reviews. The search was updated in September 2018, and an additional 142 articles were identified (of which 19 were included). A total of 275 studies were included in the review.

### Study characteristics

Out of the 275 included articles, 164 (60%) were outbreak investigations, 70 (25%) focused on strategy-oriented surveillance, and 41 (15%) on control-oriented surveillance. Almost all studies (274 out of 275) applied NGS technologies in the context of WGS, which will consequently be the focus for the remainder of this review. Table 1 gives an overview of the general characteristics of the included studies. Table 2 gives a deeper insight into the different study aims. The completed extraction form describing the characteristics of all included studies is available in the additional material [see Additional file 7].

**Table 1:** Characteristics of included studies (Jan 2015 to Sep 2018: *n* = 275)

Study characteristic	No. of studies (Jan 2015 to Sep 2018)		
	Outbreak investigations (*n* = 164)	Control-oriented surveillance (*n* = 41)	Strategy-oriented surveillance (*n* = 70)
Country^¥^,*			
USA and Canada	42	7	16
UK and Ireland	33	13	15
Australia and New Zealand	19	3	7
Germany	14	2	4
Denmark	9	4	0
France	5	2	2
Setting^¥^			
Community	94	23	47
Institutional (hospital, school, nursery, etc.)	73	21	25
Time orientation of NGS analyses^¥^			
Retrospective	97	18	59
Quasi-real time	58	0	0
Prospective	11	25	11
Level of implementation of NGS analyses^¥^			
Proof-of-concept	57	27	8
Used to address a specific public health problem	100	10	61
Implemented into routine public health	14	6	2
Sampling fraction of NGS analyses			
All available samples	57	25	21
Subset of available samples (complementary)	107	16	48
Pathogens^¥^			
*Staphylococcus aureus*	15	10	13
Multidrug-resistant Gram-negative bacteria	27	4	12
Vancomycin-resistant Enterococci (VRE)	5	6	0
*Clostridium difficile*	5	1	2
*Streptococcus* spp.	8	0	6
*Listeria monocytogenes*	13	4	1
*Shigella* spp.	1	2	2
*Salmonella* spp.	33	5	8
Shiga toxin-producing *Escherichia coli* (STEC)	10	4	1
*Campylobacter jejuni*	5	0	0
*Legionella* spp.	10	0	1
*Mycobacterium* spp.	14	6	5
*Neisseria* spp.	6	0	14
Others	13	1	6

^¥^One study can be assigned to multiple categories

*Non-exhaustive list

**Table 2:** Study aims (applications of NGS) of included studies (Jan 2015 to Sep 2018: *n* = 275)

Study aim(s)	
Outbreak investigations (*n* = 164)	
Source tracing	78
Identify transmission routes	85
Inform outbreak management: feedback on key phenotypic attributes	11
Control-oriented surveillance (*n* = 41)	
Understand transmission dynamics (identify transmission networks/clusters)	15
Early outbreak detection	23
Overview of circulating strains to identify the emergence of new threats	12
Strategy-oriented surveillance (*n* = 70)	
Understand transmission dynamics to develop prevention strategies	21
Overview of circulating strains (long-term trends)	38
Impact assessment of prevention and control programs	29
Identification of risk factors and risk groups	6

One study can have multiple study aims

Outbreak investigations of food- and waterborne pathogens mainly focused on source tracing (*n* = 78, 48%), in order to identify and eliminate the source as quickly as possible. In case of person-to-person transmission, the outbreak investigations focused on understanding transmission dynamics and reveal the spread of the pathogen in the population, in order to interrupt transmission chains and to prevent its further spread (*n* = 85, 52%). Eleven studies (7%) reported the use of WGS to provide feedback on key phenotypic attributes, such as virulence genes or antibiotic resistance, in order to inform outbreak management. The majority of the outbreak investigations were performed retrospectively (*n* = 97, 59%), i.e., as a proof-of-concept and/or to improve future preparedness by addressing a specific public health problem from the past. Fifty-eight studies (35%) applied WGS in quasi-real time, i.e., directly impacting the ongoing outbreak. In the majority of outbreak investigations, WGS was used on a subset of available samples (*n* = 107, 65%), for example, to further differentiate between isolates assigned to the same subtype as identified by conventional characterization methods. Outbreak investigations using WGS were mainly applied to *Staphylococcus aureus* (*n* = 12) and multidrug-resistant (MDR) Gram-negative bacteria (*n* = 27) in a hospital setting, to *Salmonella* spp. (*n* = 33) and *Listeria monocytogenes* (*n* = 13) during foodborne outbreaks, and to *Mycobacterium tuberculosis* (*n* = 14).

Classifying studies as control-oriented was mainly based on the fact that these studies aimed to detect events that require immediate action (e.g., early outbreak detection) or that the study was initiated following a specific public health problem. They were performed retrospectively (*n* = 18, 44%), i.e., as a proof-of-concept, and/or prospectively (*n* = 25, 61%). Six studies (15%) reported a nation-wide implementation of prospective genotyping into routine public health practices.

Strategy-oriented studies are in general conducted over larger time periods and geographical areas, in order to better understand the behavior of a certain pathogen within a population, and to plan future prevention and control programs. Twenty-nine studies (41%) applied WGS to assess the impact of prevention and control programs, mostly to evaluate vaccination programs. Genomic-informed strategy-oriented surveillance has also been frequently applied to monitor long-term changes over a larger geographic and population scale (*n* = 38, 54%) to detect the emergence of strains with enhanced virulence, to monitor drug resistance, to detect cross-border transmission events, or to identify zoonotic pathogens. Studies describing the use of WGS for strategy-oriented surveillance were often performed retrospectively (*n* = 59, 84%) on a historical subset of samples in order to answer a specific public health question.

For control- and strategy-oriented surveillance activities, WGS was mainly applied to *S. aureus* (*n* = 23), MDR Gram-negative bacteria (*n* = 16), *Neisseria meningitidis* (*n* = 14), *Salmonella* spp. (*n* = 13), and *M. tuberculosis* (*n* = 9).

### Results of individual studies

#### Outbreak investigations

WGS provides increased resolution for case ascertainment and linking possible sources to these cases during outbreak investigations of food- and waterborne pathogens compared to conventional typing methods where only a small fraction of the genome is used (e.g., pulsed-field gel electrophoresis [PFGE], multiple-locus variable-number tandem-repeat analysis [MLVA], and multi-locus sequence typing [MLST]). The discriminatory power of WGS allows heterogeneous clusters of isolates, often indistinguishable with these previously used typing methods, to be split up into smaller groups of cases that are more likely to originate from a common source [21–31]. The increased resolution of WGS is particularly useful for clonal pathogens or serotypes that show little genetic variation [32–35]. Investigations applying WGS during the course of an outbreak were able to identify the likely source of infection and rapidly implement control measures to stop further spread [36–42]. On several occasions, WGS data combined with epidemiological investigations enabled food authorities to intervene based on strong evidence and the subsequent timely recall of potentially contaminated food [43–45]. Moreover, the digital and universal nature of WGS allows data to be exchanged and analyzed between different countries during multi-national outbreaks [34, 44, 46–49]. However, this requires internationally standardized protocols and nomenclature, as well as meaningful interpretation guidelines [32]. In addition to rapid source tracing, WGS provides insights into the virulome of certain pathogenic clades [50–53]. For example, real-time WGS is able to generate timely information concerning the presence of virulence genes during a Shiga toxin-producing *Escherichia coli* (STEC) outbreak [51]. WGS was often used to guide nosocomial outbreak investigations. It was reported several times that transmission events that were suspected based on epidemiological data alone or using low-resolution strain typing methods like antibiogram profiles had been disproved by integrating WGS data [54–57]. The ability to quickly exclude a patient or potential source during an outbreak investigation is equally important for infection control purposes as the confirmation of related isolates [39], thereby preventing inappropriate, costly, and ineffective control measures [55, 56].

The most highlighted issue is the fact that WGS, as is equally the case for conventional typing methods, cannot stand on its own and that epidemiological data (including time, place, and exposure data) should complement the WGS results to identify a common source or link cases during outbreak investigations [24, 58–64]. False conclusions could be drawn from WGS data alone since it is possible that epidemiologically unrelated isolates are highly similar at the SNP level [58, 65–67]. Another reported issue was the potential misinterpretation of isolate relationships given the diversity of isolates that can be found within a single host (e.g., following long term carriage) or environmental reservoir. It was stressed by several studies that it is important to account for this “cloud of diversity” by increasing the number of samples taken from the suspected source [55, 66–73]. On the other hand, within-host diversity allows to identify long-term carriers [74].

#### Control-oriented surveillance

Several public health agencies launched pilot projects to implement WGS in routine practice for control-oriented surveillance purposes, such as early outbreak detection, and evaluated its performance [75–77]. In 2013, a multi-agency collaboration prospectively performed WGS on all available *L. monocytogenes* isolates collected from patients, food, and food processing environments in the USA. Implementation of WGS data into their surveillance activities led to the detection of an increased number of outbreak clusters. In addition, combining WGS data with robust epidemiological information solved more outbreaks compared to before with PFGE [77]. Retrospective comparisons during the transition period from traditional to WGS-based characterization were not considered an obstacle given the possibility to accurately extract traditional typing information for *L. monocytogenes* from WGS data [75]. Also for STEC O157, the added value of implementing WGS as a tool to inform national surveillance was demonstrated as it led to early and accurate outbreak detection [78], as well as the ability to extract information concerning important virulence determinants and monitor the emergence of hyper-virulent strains [79, 80]. Similarly, a prospective trial of sequencing all *Salmonella* Typhimurium isolates concurrently with the conventional MLVA typing technique in Australia demonstrated the higher resolution offered by WGS leading to better source attributions and more targeted epidemiological investigations [81]. However, several challenges related to the interpretation of WGS data remain. As for outbreak investigations, it was reported several times that WGS results should not be interpreted on their own. A single cutoff of the number of SNPs to assess relatedness cannot consistently predict whether isolates are epidemiologically linked [77, 81–83]. However, field data (e.g., demographic data or exposure histories) are only valuable when organized in a standardized format, requiring a more systematic approach to epidemiological data collection [84]. Another implementation barrier reported was the limited capacity of the public health unit to understand and use WGS data, implying an increased need for collaboration and exchange of expertise between microbiologists, bioinformaticians, and epidemiologists [81].

NGS has been applied to monitor antimicrobial resistance of hospital-acquired infections. Genotypic prediction of resistance of *S. aureus* strains seems at least as reliable as routine phenotypic testing. However, phenotypic prediction based on the genotype cannot replace phenotypic testing, as the present understanding of the genetic basis of resistance and the associated databases are not comprehensive [85]. This limitation was shown during a WGS-based surveillance of antimicrobial resistant determinants in *Klebsiella pneumonia* where the phenotypically determined resistance was higher than the sequenced-based resistance [86].

WGS has proven to be a more reliable tool to predict epidemiological links between tuberculosis cases than the conventional variable number of tandem repeat (VNTR) genotyping that often lead to false cluster identification [87]. WGS as a tool for the identification of tuberculosis outbreaks may be particularly useful in settings where the genetic diversity is expected to be lower such as geographically restricted *M. tuberculosis* populations [88], genetically closely related genotypes imported from a high-incidence region [89], or for highly monomorphic *M. tuberculosis* lineages [90].

#### Strategy-oriented surveillance

Several studies showed the value of WGS in understanding the impact of vaccination on circulating pathogen populations, potentially resulting in antigenic drift to escape vaccine-mediated immune selective pressure (i.e., strain replacement) [91–100]. Gaining insights into this is achieved by comparing the incidence of infections caused by vaccine targeted serotypes before and after the introduction of the vaccine, and to potentially identify the proliferation of non-vaccine targeted strains. The adoption of WGS methods to monitor pathogen populations during immunization programs has proven to be useful and could potentially identify differential impacts on distinct serotypes [91]. Genomic surveillance provides the required resolution for the development of targeted interventions [92] and to predict the impact of implementing a vaccination program in a given population [101]. The routine use of WGS for surveillance purposes can also inform antibiotic stewardship. One advantage of introducing WGS to inform treatment guidelines is the ability to identify genetically linked resistance that can be co-selected by multiple drugs, as opposed to phenotypic resistance rates that consider each antimicrobial class as a discrete unit [102]. In addition, resistance rates can vary significantly by clone implying that monitoring changes in population structure using WGS is useful to guide antibiotic usage policies [103]. Besides informing vaccination programs and antibiotic stewardship, WGS can reveal a detailed understanding of the transmission dynamics within and between healthcare settings, the community, and individual households, to appropriately direct control programs and decolonization strategies [104–112].

Several studies highlighted the public health benefits of WGS-guided surveillance to monitor the spread of multidrug-resistant isolates and mobile genes, including resistance-carrying transposons and plasmids that are able to transfer resistance between bacterial species [113–118]. The zoonotic potential of clinically relevant multi-resistant bacteria stresses the importance of the ‘One Health’ approach. In general, WGS-informed surveillance including isolates from different hosts and settings can provide evidence for interspecies transmission and focus control efforts on important reservoirs [119–126].

### Reported challenges, issues, and obstacles from an epidemiologist’s perspective

Several studies reported challenges, issues, and obstacles (not exclusively) related to the integration of pathogen genomics within the activities of epidemiologists. For example, the inability to link laboratory and contextual data due to missing unique identifiers [127] impedes proper data integration. Further, contextual data was often missing, limited, or unstandardized [30, 34, 38, 49, 62, 71, 84, 94, 108, 128–140]. Regarding the sampling strategy, selection bias might arise when WGS has been performed on a small proportion of cases/isolates, severe cases are overrepresented among sequenced isolates, asymptomatic cases/carriers are excluded, or certain geographical regions or time periods are overrepresented [24, 30, 32, 52, 59, 85, 91, 93, 106, 116, 128, 141–144]. In addition, there might be insufficient statistical power to detect associations due to a low number of sequenced strains [81, 128, 144, 145].

## Discussion

### Applications

Within the set of studies included in this scoping review, NGS was mainly used as a tool to provide information on the whole genome of the bacterial pathogens. WGS has useful applications in both outbreak investigations and surveillance activities. Outbreak investigations benefit from the increased resolution offered by WGS for case ascertainment, linking cases to the possible sources, defining transmission clusters, and providing rapid feedback on key phenotypic attributes of the involved pathogens. The application of WGS during control-oriented surveillance was mainly aimed at early outbreak detection by accurately defining transmission clusters among circulating strains, unraveling transmission chains to guide targeted interventions, and identifying the emergence of new threats. The use of WGS during strategy-oriented surveillance seemed particularly useful to assess the impact of prevention and control programs, such as vaccination campaigns and antibiotic stewardship.

### Level of implementation

WGS has been increasingly used as a typing tool for comparison of isolates during outbreak investigations. Most published studies were retrospective (59%), but an increasing number applied WGS in quasi-real time. For surveillance activities of certain pathogens, there has been a shift from proof-of-concept studies to routine use of WGS. In several countries, public health agencies and regulatory bodies [e.g., Public Health England, US Centers for Disease Prevention and Control (CDC), European Food Safety Authority (EFSA)] have implemented WGS as a routine typing tool for surveillance activities of selected pathogens. Although WGS is (or was) mainly used in parallel with conventional testing in many European countries [76, 81, 82, 87, 146], countries such as Denmark, France, and the UK have already transitioned completely to WGS for certain pathogens [34, 43, 46, 76, 84, 147–150]. Following the results of a survey conducted by ECDC, 20 countries (i.e., two thirds of European Union and European Economic Area countries) were routinely using WGS in 2017 for national surveillance of at least one human pathogen [151].

### Added value

WGS has shown superior sensitivity and specificity to identify transmission clusters compared to traditional subtyping methods such as PFGE, MLVA, and MLST that often do not provide the required resolution to discriminate between outbreak-related and sporadic cases [21–31, 59]. Thanks to its greater specificity, WGS allows to reject a false hypothesis of transmission generated by conventional methods, thereby avoiding inappropriate, costly, and ineffective follow-up investigations and control measures [39, 55, 56, 78, 152]. More targeted interventions can save resources at the health protection and local authority level [78]. The major advantage of implementing WGS during surveillance activities or outbreak investigations is therefore inherent in the higher resolution of the WGS output itself. It should be noted that the utility of WGS varies depending on the public health objective (discriminating between closely related individual cases during a point-source outbreak or national surveillance purposes) [153], as well as on the population structure (high incidence settings versus low-transmission settings) [88, 89] and the clonality of the pathogen [32–35, 90]. Also, a stepwise implementation of typing methods has proven to be a useful approach. Conventional molecular methods can serve as a first-level classification to confine possible outbreak isolates. At the next level, WGS can bring deeper and more comprehensive insights [130, 154].

In terms of technical advantages, WGS is a universal test that is applicable to all organisms [155] and has the potential to provide multiple tests in silico (e.g., antibiotic resistance, serotype, virulence genes) from a single assay, thereby replacing several conventional methods and/or providing additional information on the studied pathogen [57, 79, 156, 157]. Therefore, NGS is able to replace current time-consuming and labor-intensive methods with a single, all-inclusive diagnostic test [94, 157, 158]. Moreover, the digital nature and the reliability of WGS data allow exchange and to compare data across countries [44, 46, 47]. The development of shared databases will make it increasingly possible to establish links between sequences from different countries and sources.

### Challenges, issues, and obstacles

#### Definition of a cluster

The main issue reported when using WGS data to detect and to confirm transmission between isolates was the difficulty, if not impossibility, to define with a single SNP/allele threshold how much genetic variation can exist within an epidemiologically related cluster [1, 23, 28, 57, 58, 65, 67, 159]. The number of SNPs within a cluster often depends on various factors, such as the genetic diversity within each species, its molecular clock, evolutionary forces, the nature of the outbreak (point-source, long-lasting, multinational, etc.), the extent of diversity in the background population, within-host diversity, the population bottleneck during transmission, the level of asymptomatic infections, the number of isolates included in the analysis, and the methods used for genomic analysis [1, 25, 67, 74, 81, 83, 147, 160–162]. Many studies stress the fact that we cannot rely solely on genomic information during outbreak investigations or surveillance activities and that epidemiological data describing the temporal and spatial dynamics of infection should always be considered [59–61, 63, 67, 77, 81–83]. Contextual data should therefore be collected carefully and combined with WGS data for a proper interpretation, which is almost seamlessly linked to the challenge of data integration.

#### Data integration

A useful interpretation of genomic data is highly dependent on the epidemiological and clinical metadata [1, 160, 163, 164]. The integration of laboratory and epidemiological data is often hampered by the incomplete and/or unstructured nature of the contextual data [13, 84, 128, 165]. For example, during a multi-country outbreak investigation, it is important to develop a codebook for uniform and standardized data entry between countries [34, 49]. To maximize the potential of WGS, public health professionals have to identify a minimum set of variables (such as time, place of infection, host characteristics, clinical presentation, and exposures) that should be incorporated within surveillance activities of a particular pathogen [165].

Although WGS data has the potential to support phenotypic predictions of virulence and resistance based on the genotype, phenotypic data will still be needed to identify new resistance/virulence mechanisms and to keep the databases up-to-date [12, 85, 86, 157, 166, 167]. Therefore, phenotypic testing results and clinical data have to be collected in a standardized manner alongside the sequence data to feed the databases from which associations between genotype and phenotype can be observed [160].

More recently, digital streams (also called “Internet of things”) are being used as an input for surveillance systems (i.e., digital epidemiology). Examples include search engines, social media, mobile phones, and health trackers. These novel data streams, generated outside public health, could potentially enrich epidemiology by providing information on natural and social phenomena [168].

One Health, the concept of structured collaboration and coordination between human, animal, and eco health systems, has become an emerging focus due to the increased understanding of how animal and ecological reservoirs significantly influence human health [169]. Therefore, the management of infectious diseases requires sampling from different hosts and sources. As indicated by Rantsiou et al., the development of WGS is currently not at the same level in the food industry as compared to public health agencies [170]. The outputs produced by the different sectors should remain comparable at any time to ensure the linkage of isolates.

**Fig. 3:**
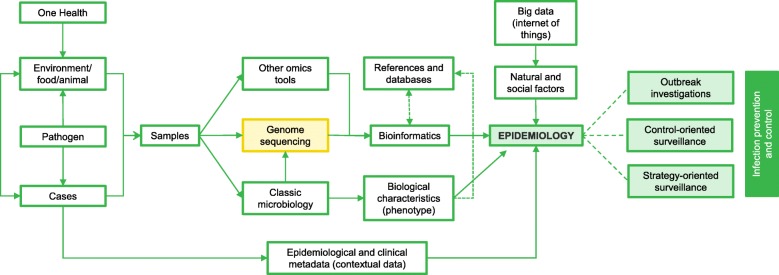
Integration of multiple data types. The anticipated workflow of infection prevention and control includes the following: (1) samples are obtained from cases infected with a certain pathogen, as well as from other sources such as the environment, food, and/or animals following the One Health approach; (2) pathogens are isolated, and information concerning the biological characteristics is obtained through classical microbiological testing. Phenotypic tests are still required to feed databases and confirm genotype-phenotype associations. Culturing steps (isolation) are often preceding genome sequencing; however, sequencing directly from clinical samples is also possible using culture-independent methods (metagenomics); (3) high-throughput sequence data is generated (other -omics technologies such as transcriptomics, proteomics, and metabolomics can complement the genomic information); (4) relationships among isolates and specific characteristics are inferred based on sequence information obtained through bioinformatics tools; (5) to come to a meaningful outcome (i.e., transmission chains, cluster identification, source tracing, key phenotypic attributes), the genomic evidence is combined with epidemiological metadata (time, place, exposures, etc.) from field epidemiological investigations, clinical data obtained through the healthcare system, biological characteristics obtained through classic microbiological methods, and big data on natural and social factors. Finally, infection prevention and control measures can be conducted on the basis of this aggregated information

An overview of data integration is presented in Fig. 3.

#### Collaboration between the different stakeholders

Following the previous section addressing the importance of data integration, it is clear that the switch to WGS requires an increase in multi-disciplinary working [9, 81, 148, 171]. In particular for data interpretation, expertise in bioinformatics and in biological, epidemiological, and microbiological sciences needs to be combined. Infectious disease epidemiologists implementing WGS data into their routine workflow might need training in genomics as well as skills in analyzing high-dimensional data sets. In addition to interdisciplinary and inter-sectoral (One Health) collaboration, the implementation of WGS should be coordinated at an international level as infectious diseases do not respect national boundaries [49].

#### Sampling frame

As for any type of epidemiological study, genomics-informed surveillance activities should be based on robust sampling strategies defining the required sample size and the number of samples needed from the different sources. The sampling framework will vary depending on the type of public health application (e.g., investigating an explosive outbreak often requires dense sampling including multiple samples from single sources, as opposed to strategy-oriented surveillance requiring a representative coverage of the population) [171]. Selection bias can be introduced when the subset of samples selected for WGS is not representative [128, 163]. In order to efficiently assemble a representative sample, it is often needed to develop a stratified sampling scheme according to time, place, and person, or to perform normalizations to maintain original sampling fractions.

#### Translational research and information overload

Genomic information must be interpreted and translated in a meaningful manner into both immediate public health action and longer term prevention programs [2, 57, 172]. Moving from low-resolution typing methods to WGS-based typing will lead to an increase in the detected number of hazards. Not only will the sensitive nature of WGS increase the number of clusters detected [76, 77], it will also provide additional information on the presence of resistance genes, virulence genes, etc. It will be important to filter out the hazards that are truly relevant from a public health point of view (i.e., separate the “signal” from the “noise”) and that subsequently require the initiation of a public health action.

### Limitations of the scoping review

A possible limitation is the fact that only studies published after 2015 were included. However, NGS is a fast-evolving technique, and we were mainly interested in its state-of-the-art applications. Another potential limitation is that only one database (PubMed) was searched. However, hand searching and reference checking were applied to partly account for this. Still, it is likely that additional publications exist outside this search. Some work will have been missed, but the aim was to have a systematic overview of the field rather than an exhaustive capture of every single published article. An encountered difficulty during the selection process was the subjective nature of the inclusion and exclusion criteria, mainly in terms of classifying the studies according to their context (public health, research, or diagnostics). We tried to account for this by screening the full texts by two independent reviewers. Further, data extraction was based only on the data provided in the individual studies and, if available, supplementary information. Although the data extraction was performed using a standard procedure, it is possible that some information was misinterpreted. Given the large number of included studies, it was opted not to contact the investigators to retrieve additional data.

### Future perspectives

Currently, the most common strategy to integrate WGS into routine public health surveillance is to add genomic typing to conventional surveillance activities where an increased resolution is considered necessary, i.e., on a selected subset of isolates. As costs will drop, microbiology laboratories will potentially implement WGS in their routine workflow. Following this scenario, the use of WGS is foreseen for all clinical isolates and the number of isolates sequenced would no longer be driven by pre-determined study designs [157, 163]. This way, sequence data gathered for diagnostic purposes can be accumulated for public health activities. In addition, large-scale research into genotype-phenotype associations from routinely collected data will be possible [157].

## Conclusions

This scoping review addresses the current state and potential of implementing pathogen genomics for routine public health practice. Main applications include the use of WGS data for (1) source tracing during outbreak investigations, (2) early outbreak detection, (3) unraveling transmission dynamics in order to implement targeted interventions, (4) monitoring drug resistance, (5) detecting cross-border transmission events, (6) identifying the emergence of strains with enhanced virulence or strains with zoonotic potential, and (7) assessing the impact of prevention and control programs, such as vaccination campaigns. The main reported added value of WGS by the included studies is the superior resolution compared to the conventional methods, and consequently being able to accurately confirm or rule out transmission events. However, it should be emphasized that WGS cannot stand on its own and should be integrated with other data types. High-quality epidemiological data and study designs are needed to realize the full potential of WGS. Collaborations between infectious disease epidemiologists, public health practitioners, microbiologists, and bioinformaticians are key for a successful genomics-informed surveillance.

## Additional files

Additional file 1: PRISMA checklist. Completed PRISMA checklist indicating page number in manuscript of relevant content. (PDF 409 kb)

Additional file 2: Protocol. (PDF 939 kb)

Additional file 3: Inclusion and exclusion criteria organized according to the Population-Concept-Context (PCC) principle, as outlined in the Joanna Briggs’ Manual for Scoping Reviews. (PDF 499 kb)

Additional file 4: Decision tree used during screening to distinguish between research, individual patient care, and public health practice. (PDF 275 kb)

Additional file 5: PubMed search string. (PDF 176 kb)

Additional file 6: Extraction form listing the characteristics that are extracted from the included studies. (PDF 179 kb)

Additional file 7: Characteristics of included studies.(XLSX 105 kb)

## Data Availability

The dataset(s) supporting the conclusions of this article is (are) included within the article (and its additional file(s)).

## Abbreviations

CDC: US Centers for Disease Prevention and Control
ECDC: European Centre for Disease Prevention and Control
EEA: European Economic Area
EFSA: European Food Safety Authority
EU: European Union
MDR: Multidrug-resistant
MLST: Multi-locus sequence typing
MLVA: Multiple-locus variable-number tandem-repeat analysis
NGS: Next-generation sequencing
PFGE: Pulsed-field gel electrophoresis
PRISMA: Preferred Reporting Items for Systematic Reviews and Meta-Analyses
SNP: Single-nucleotide polymorphism
STEC: Shiga toxin-producing *Escherichia* coli
VNTR: Variable number of tandem repeat
VRE: Vancomycin-resistant Enterococci
WGS: Whole-genome sequencing

## References

[1] Kan B, Zhou H, Du P, Zhang W, Lu X, Qin T (2018). Transforming bacterial disease surveillance and investigation using whole-genome sequence to probe the trace. Front Med.

[2] Tang P, Croxen MA, Hasan MR, Hsiao WWL, Hoang LM (2017). Infection control in the new age of genomic epidemiology. Am J Infect Control.

[3] Struelens MJ, Brisse S. From molecular to genomic epidemiology: transforming surveillance and control of infectious diseases. Eurosurveillance. 2013;18(4):pii = 20386. Available from: http://www.eurosurveillance.org/ViewArticle.aspx?ArticleId = 20386 10.2807/ese.18.04.20386-en 23369387

[4] Gwinn M, MacCannell RD, Khabbaz FR (2017). Integrating advanced molecular technologies into public health. J Clin Microbiol.

[5] Besser J, Carleton HA, Gerner-Smidt P, Lindsey RL, Trees E (2018). Next-generation sequencing technologies and their application to the study and control of bacterial infections. Clin Microbiol Infect.

[6] Baker MG, Easther S, Wilson N. A surveillance sector review applied to infectious diseases at a country level. BMC Public Health. 2010. 10.1186/1471-2458-10-332. 10.1186/1471-2458-10-332 PMC3224743 20540772

[7] Palmer SR (1989). Review article: Epidemiology in search of infectious diseases: methods in outbreak investigation. J Epidemiol Community Health.

[8] Thacker SB, Berkelman RL (1988). Public health surveillance in the United States. Epidemiol Rev.

[9] Muellner P, Pleydell E, Pirie R, Baker MG, Campbell D, Carter PE, et al. Molecular-based surveillance of campylobacteriosis in New Zealand - from source attribution to genomic epidemiology. Eurosurveillance. 2013;18(3):pii = 20365. Available from: http://www.eurosurveillance.org/ViewArticle.aspx?ArticleId = 20365 23351655

[10] European Centre for Disease Prevention and Control. ECDC roadmap for integration of molecular and genomic typing into European-level surveillance and epidemic preparedness – Version 2.1, 2016-19. 2016. Available from: https://ecdc.europa.eu/sites/portal/files/media/en/publications/Publications/molecular-typing-EU-surveillance-epidemic-preparedness-2016-19-roadmap.pdf

[11] Kwong JC, Mccallum N, Sintchenko V, Howden BP (2015). Whole genome sequencing in clinical and public health microbiology. Pathology.

[12] Goldberg B, Sichtig H, Geyer C, Ledeboer N, Weinstock GM. Making the leap from research laboratory to clinic: challenges and opportunities for Next-Generation Sequencing in infectious disease diagnostics. MBio. 2015;6(6). 10.1128/mBio.01888-15. 10.1128/mBio.01888-15 PMC4669390 26646014

[13] MacCannell D (2016). Next generation sequencing in clinical and public health microbiology. Clin Microbiol Newsl.

[14] Deurenberg RH, Bathoorn E, Chlebowicz MA, Couto N, Ferdous M, García-Cobos S (2017). Application of next generation sequencing in clinical microbiology and infection prevention. J Biotechnol.

[15] Loman NJ, Pallen MJ (2015). Twenty years of bacterial genome sequencing. Nat Rev Microbiol.

[16] Fournier P-E, Dubourg G, Raoult D. Clinical detection and characterization of bacterial pathogens in the genomics era. Genome Med. 2014. 10.1186/s13073-014-0114-2. 10.1186/s13073-014-0114-2 PMC4295418 25593594

[17] Grant MJ, Booth A (2009). A typology of reviews: an analysis of 14 review types and associated methodologies. Health Info Libr J.

[18] Moher D, Liberati A, Tetzlaff J, Altman DG, Antes G, Atkins D (2009). Preferred reporting items for systematic reviews and meta-analyses: the PRISMA statement. PLoS Med.

[19] Peters MDJ, Godfrey C, McInerney P, Baldini Soares C, Khalil H, Parker D. Chapter 11: Scoping Reviews. Aromataris E, Munn Z (Editors). Joanna Briggs Institute Reviewer's Manual. The Joanna Briggs Institute. 2017. Available from https://reviewersmanual.joannabriggs.org/.

[20] Arksey H, O’Malley L (2005). Scoping studies: towards a methodological framework. Int J Soc Res Methodol Theory Pract.

[21] Bekal S, Berry C, Reimer AR, Van Domselaar G, Beaudry G, Fournier E (2016). Usefulness of high-quality core genome single-nucleotide variant analysis for subtyping the highly clonal and the most prevalent *Salmonella enterica* Serovar heidelberg clone in the context of outbreak investigations. J Clin Microbiol.

[22] Vincent C, Usongo V, Berry C, Tremblay DM, Moineau S, Yousfi K (2018). Comparison of advanced whole genome sequence-based methods to distinguish strains of *Salmonella enterica* serovar Heidelberg involved in foodborne outbreaks in Québec. Food Microbiol.

[23] Chen Y, Luo Y, Pettengill J, Timme R, Melka D, Doyle M (2017). Singleton sequence type 382, an emerging clonal group of *Listeria monocytogenes* associated with three multistate outbreaks linked to contaminated stone fruit, caramel apples, and leafy green salad. J Clin Microbiol.

[24] Taylor AJ, Lappi V, Wolfgang WJ, Lapierre P, Palumbo MJ, Medus C (2015). Characterization of foodborne outbreaks of *Salmonella enterica* serovar enteritidis with whole-genome sequencing single nucleotide polymorphism-based analysis for surveillance and outbreak detection. J Clin Microbiol.

[25] Wuyts V, Denayer S, Roosens NHC, Mattheus W, Bertrand S, Marchal K, et al. Whole genome sequence analysis of salmonella enteritidis PT4 outbreaks from a national reference laboratory’s viewpoint. PLoS Curr. 2015. 10.1371/currents.outbreaks.aa5372d90826e6cb01. 10.1371/currents.outbreaks.aa5372d90826e6cb0136ff66bb7a62fc PMC4593640 26468422

[26] Wilson MR, Brown E, Keys C, Strain E, Luo Y, Muruvanda T (2016). Whole genome DNA sequence analysis of *Salmonella* subspecies *enterica* serotype Tennessee obtained from related peanut butter foodborne outbreaks. PLoS One.

[27] Chen Y, Luo Y, Curry P, Timme R, Melka D, Doyle M (2017). Assessing the genome level diversity of *Listeria monocytogenes* from contaminated ice cream and environmental samples linked to a listeriosis outbreak in the United States. PLoS One.

[28] Dahl Viktor, Sundqvist Lena, Hedenström Ingela, Löfdahl Margareta, Alm Erik, Ringberg Håkan, Lindblad Mats, Wallensten Anders, Thisted Lambertz Susanne, Jernberg Cecilia (2017). A nationwide outbreak of listeriosis associated with cold-cuts, Sweden 2013-2014. Infection Ecology & Epidemiology.

[29] Raphael BH, Baker DJ, Nazarian E, Lapierre P, Bopp D, Kozak-muiznieks NA (2016). Genomic resolution of outbreak-associated *Legionella pneumophila* Serogroup 1 Isolates from New York State. Appl Environ Microbiol.

[30] Yacisin K, Hsieh JL, Weiss D, Ackelsberg J, Lee E, Jones L (2017). Outbreak of non-tuberculous mycobacteria skin or soft tissue infections associated with handling fish - New York City, 2013-2014. Epidemiol Infect.

[31] Crowe SJ, Green A, Hernandez K, Peralta V, Bottichio L, Defibaugh-Chavez S (2017). Utility of combining whole genome sequencing with traditional investigational methods to solve foodborne outbreaks of Salmonella infections associated with chicken: a new tool for tackling this challenging food vehicle. J Food Prot.

[32] Simon S, Trost E, Bender J, Fuchs S, Malorny B, Rabsch W (2018). Evaluation of WGS based approaches for investigating a food-borne outbreak caused by *Salmonella enterica* serovar Derby in Germany. Food Microbiol.

[33] Hassan R, Tecle S, Adcock B, Kellis M, Weiss J, Saupe A (2018). Multistate outbreak of *Salmonella* Paratyphi B variant L(+) tartrate(+) and *Salmonella* Weltevreden infections linked to imported frozen raw tuna: USA, March-July 2015. Epidemiol Infect.

[34] Inns T, Ashton PM, Herrera-Leon S, Lighthill J, Foulkes S, Jombart T (2017). Prospective use of whole genome sequencing (WGS) detected a multi-country outbreak of *Salmonella* Enteritidis. Epidemiol Infect.

[35] Kuijpers F, Le Hello S, Fawal N, Fabre L, Tourdjman M, Dufour M, et al. Genomic analysis of *Salmonella enterica* serotype Paratyphi A during an outbreak in Cambodia , 2013 – 2015. Microb Genomics. 2016;2(11):e000092. 10.1099/mgen.0.000092 PMC5320704 28348832

[36] Hassan R, Rounds J, Sorenson A, Leos G, Concepción-Acevedo J, Griswold T (2017). Multistate outbreak of *Salmonella* anatum infections linked to imported hot peppers — United States, May–July 2016. Morb Mortal Wkly Rep.

[37] Mair-Jenkins J, Borges-Stewart R, Harbour C, Cox-Rogers J, Dallman T, Ashton P, et al. Investigation using whole genome sequencing of a prolonged restaurant outbreak of Salmonella Typhimurium linked to the building drainage system, England, February 2015 to March 2016. Euro Surveill. 2015;22(49):pii = 17-00037. Available from: 10.2807/1560-7917.ES.2017.22.49.17-00037 10.2807/1560-7917.ES.2017.22.49.17-00037 PMC5727591 29233257

[38] Angelo KM, Chu A, Anand M, Nguyen T-A, Bottichio L, Wise M (2015). Outbreak of *Salmonella* newport infections linked to cucumbers - United States, 2014. Morb Mortal Wkly Rep.

[39] Davis RJ, Jensen SO, Van Hal S, Espedido B, Gordon A, Farhat R (2015). Whole genome sequencing in real-time investigation and management of a *Pseudomonas aeruginosa* outbreak on a neonatal intensive care unit. Infect Control Hosp Epidemiol.

[40] Lapierre P, Nazarian E, Zhu Y, Wroblewski D, Saylors A, Passaretti T (2017). Legionnaires’ disease outbreak caused by endemic strain of *Legionella pneumophila*, New York, New York, USA, 2015. Emerg Infect Dis.

[41] Quick J, Ashton P, Calus S, Chatt C, Gossain S, Hawker J, et al. Rapid draft sequencing and real-time nanopore sequencing in a hospital outbreak of *Salmonella*. Genome Biol. 2015;16(114):doi:10.1186/s13059-015-0677-2. Available from: 10.1186/s13059-015-0677-2 10.1186/s13059-015-0677-2 PMC4702336 26025440

[42] Weiss D, Boyd C, Rakeman JL, Greene SK, Fitzhenry R, McProud T (2017). A large community outbreak of Legionnaires’ disease associated with a cooling tower in New York City, 2015. Public Health Rep.

[43] Gillesberg Lassen S, Ethelberg S, Björkman JT, Jensen T, Sørensen G, Kvistholm Jensen A (2016). Two listeria outbreaks caused by smoked fish consumption—using whole-genome sequencing for outbreak investigations. Clin Microbiol Infect.

[44] Schjørring S, Gillesberg Lassen S, Jensen T, Moura A, Kjeldgaard JS, Müller L, et al. Cross-border outbreak of listeriosis caused by cold-smoked salmon, revealed by integrated surveillance and whole genome sequencing (WGS), Denmark and France, 2015 to 2017. Eurosurveillance. 2017;22(50):pii = 17-00762. Available from: 10.2807/1560-7917.ES.2017.22.50.17-00762. 10.2807/1560-7917.ES.2017.22.50.17-00762 PMC5743096 29258647

[45] Self JL, Conrad A, Stroika S, Jackson A, Burnworth L, Beal J (2016). Notes from the field: outbreak of listeriosis associated with consumption of packaged salad - United States and Canada, 2015-2016. Morb Mortal Wkly Rep.

[46] Dallman T, Inns T, Jombart T, Ashton P, Loman N, Chatt C (2016). Phylogenetic structure of European *Salmonella* Enteritidis outbreak correlates with national and international egg distribution network. Microb Genomics.

[47] Fonteneau L, Jourdan Da Silva N, Fabre L, Ashton P, Torpdahl M, Müller L, et al. Multinational outbreak of travel-related *Salmonella* chester infections in europe, summers 2014 and 2015. Eurosurveillance. 2017;22(7):pii = 30463. Available from: 10.2807/1560-7917.ES.2017.22.7.3046 10.2807/1560-7917.ES.2017.22.7.30463 PMC5322187 28230522

[48] Inns T, Lane C, Peters T, Dallman T, Chatt C, McFarland N, et al. A multi-country *Salmonella* Enteritidis phage type 14b outbreak associated with eggs from a German producer: “near real-time” application of whole genome sequencing and food chain investigations, United Kingdom, may to september 2014. Eurosurveillance. 2015;20(16):pii = 21098. Available from: http://www.eurosurveillance.org/ViewArticle.aspx?ArticleId = 21098 10.2807/1560-7917.es2015.20.16.21098 25953273

[49] Pärn T, Dahl V, Lienemann T, Perevosčikovs J, De Jong B (2017). Multi-country outbreak of *Salmonella* enteritidis infection linked to the international ice hockey tournament. Epidemiol Infect.

[50] Butcher H, Elson R, Chattaway MA, Featherstone CA, Willis C, Jorgensen F (2016). Whole genome sequencing improved case ascertainment in an outbreak of Shiga toxin-producing *Escherichia coli* O157 associated with raw drinking milk. Epidemiol Infect.

[51] Moran-Gilad J, Rokney A, Danino D, Ferdous M, Alsana F, Baum M (2017). Real-time genomic investigation underlying the public health response to a Shiga toxin-producing *Escherichia coli* O26:H11 outbreak in a nursery. Epidemiol Infect.

[52] Usein CR, Ciontea AS, Militaru CM, Condei M, Dinu S, Oprea M, et al. Molecular characterisation of human Shiga toxin-producing *Escherichia coli* O26 strains: results of an outbreak investigation, Romania, february to august 2016. Eurosurveillance. 2017;22(47):pii = 17-00148. 10.2807/1560-7917.ES.2017.22.47.17-00148 PMC5710660 29183554

[53] Oakeson KF, Wagner JM, Rohrwasser A, Atkinson-Dunn R. Whole genome sequencing and bioinformatic analysis of two foodborne illness outbreaks: *Campylobacter jejuni* and *Salmonella enterica*. J Clin Microbiol. 2018. 10.1128/JCM.00161-18. 10.1128/JCM.00161-18 PMC6204689 30158193

[54] Azarian T, Cook RL, Johnson JA, Guzman N, McCarter YS, Gomez N (2015). Whole-genome sequencing for outbreak investigations of Methicillin-Resistant *Staphylococcus aureus* in the neonatal intensive care unit: time for routine practice?. Infect Control Hosp Epidemiol.

[55] Roe CC, Horn KS, Driebe EM, Bowers J, Terriquez JA, Keim P, et al. Whole genome SNP typing to investigate methicillin-resistant *Staphylococcus aureus* carriage in a health-care provider as the source of multiple surgical site infections. Hereditas. 2016. 10.1186/s41065-016-0017-x. 10.1186/s41065-016-0017-x PMC5226111 28096773

[56] Hughes HY, Conlan SP, Lau AF, Dekker JP, Michelin AV, Youn JH (2016). Detection and whole-genome sequencing of carbapenemase-producing *Aeromonas hydrophila* isolates from routine perirectal surveillance culture. J Clin Microbiol.

[57] Durand G, Javerliat F, Bes M, Veyrieras JB, Guigon G, Mugnier N, et al. Routine whole-genome sequencing for outbreak investigations of *Staphylococcus aureus* in a national reference center. Front Microbiol. 2018. 10.3389/fmicb.2018.00511. 10.3389/fmicb.2018.00511 PMC5869177 29616014

[58] Ashton PM, Peters T, Ameh L, McAleer R, Petrie S, Nair S, et al. Whole genome sequencing for the retrospective investigation of an outbreak of *Salmonella* Typhimurium DT 8. PLoS Curr. 2015. 10.1371/currents.outbreaks.2c05a47d292f376afc. 10.1371/currents.outbreaks.2c05a47d292f376afc5a6fcdd8a7a3b6 PMC4336196 25713745

[59] Thompson CK, Wang Q, Bag SK, Franklin N, Shadbolt CT, Howard P (2017). Epidemiology and whole genome sequencing of an ongoing point-source *Salmonella* Agona outbreak associated with sushi consumption in western Sydney, Australia 2015. Epidemiol Infect.

[60] Hoffmann M, Luo Y, Monday SR, Gonzalez-Escalona N, Ottesen AR, Muruvanda T (2016). Tracing origins of the *Salmonella* Bareilly strain causing a food-borne outbreak in the United States. J Infect Dis.

[61] Barkley JS, Gosciminski M, Miller A (2016). Whole-genome sequencing detection of ongoing *Listeria* contamination at a restaurant, Rhode Island, USA, 2014. Emerg Infect Dis.

[62] Chen Y, Luo Y, Carleton H, Timme R, Melka D, Muruvanda T (2017). Whole genome and core genome multilocus sequence typing and single nucleotide polymorphism analyses of *Listeria monocytogenes* isolates associated with an outbreak linked to cheese, United States, 2013. Appl Environ Microbiol.

[63] Fernandes AM, Balasegaram S, Willis C, Wimalarathna HML, Maiden MC, McCarthy ND (2015). Partial failure of milk pasteurization as a risk for the transmission of *Campylobacter* from cattle to humans. Clin Infect Dis.

[64] Norheim G, Seterelv S, Arnesen T, Mengshoel A, Tonjum T, Ronning JO (2017). Tuberculosis outbreak in an educational institution in Norway. J Clin Microbiol.

[65] David S, Afshar B, Mentasti M, Ginevra C, Podglajen I, Harris SR (2017). Seeding and establishment of *Legionella pneumophila* in hospitals: Implications for genomic investigations of nosocomial legionnaires’ disease. Clin Infect Dis.

[66] Rosendahl Madsen AM, Holm A, Jensen TG, Knudsen E, Lundgaard H, Skov MN (2017). Whole-genome sequencing for identification of the source in hospital-acquired Legionnaires’ disease. J Hosp Infect.

[67] Casali Nicola, Broda Agnieszka, Harris Simon R., Parkhill Julian, Brown Timothy, Drobniewski Francis (2016). Whole Genome Sequence Analysis of a Large Isoniazid-Resistant Tuberculosis Outbreak in London: A Retrospective Observational Study. PLOS Medicine.

[68] Yang S, Hemarajata P, Hindler J, Li F, Adisetiyo H, Aldrovandi G (2017). Evolution and transmission of carbapenem-resistant *Klebsiella pneumoniae* expressing the *blaOXA-232* gene during an institutional outbreak associated with endoscopic retrograde cholangiopancreatography. Clin Infect Dis.

[69] Ruppé E, Olearo F, Pires D, Baud D, Renzi G, Cherkaoui A (2017). Clonal or not clonal? Investigating hospital outbreaks of KPC-producing *Klebsiella pneumoniae* with whole-genome sequencing. Clin Microbiol Infect.

[70] McRobb E, Sarovich DS, Price EP, Kaestli M, Mayo M, Keim P (2015). Tracing melioidosis back to the source: using whole-genome sequencing to investigate an outbreak originating from a contaminated domestic water supply. J Clin Microbiol.

[71] Haller S, Eller C, Hermes J, Kaase M, Steglich M, Radonic A (2015). What caused the outbreak of ESBL-producing *Klebsiella pneumoniae* in a neonatal intensive care unit, Germany 2009 to 2012? Reconstructing transmission with epidemiological analysis and whole-genome sequencing. BMJ Open.

[72] Parcell BJ, Oravcova K, Pinheiro M, Holden MTG, Phillips G, Turton JF (2018). *Pseudomonas aeruginosa* intensive care unit outbreak: winnowing of transmissions with molecular and genomic typing. J Hosp Infect.

[73] Stucki D, Ballif M, Bodmer T, Coscolla M, Maurer AM, Droz S (2015). Tracking a tuberculosis outbreak over 21 years: strain-specific single-nucleotide polymorphism typing combined with targeted whole-genome sequencing. J Infect Dis.

[74] Gordon NC, Pichon B, Golubchik T, Wilson DJ, Paul J, Blanc DS (2017). Whole-genome sequencing reveals the contribution of long-term carriers in *Staphylococcus aureus* outbreak investigation. J Clin Microbiol.

[75] Kwong JC, Mercoulia K, Tomita T, Easton M, Li HY, Bulach DM (2016). Prospective whole-genome sequencing enhances national surveillance of *Listeria monocytogenes*. J Clin Microbiol.

[76] Moura A, Tourdjman M, Leclercq A, Hamelin E, Laurent E, Fredriksen N (2017). Real-time whole-genome sequencing for surveillance of *Listeria monocytogenes*. France. Emerg Infect Dis.

[77] Jackson BR, Tarr C, Strain E, Jackson KA, Conrad A, Carleton H (2016). Implementation of nationwide real-time whole-genome sequencing to enhance listeriosis outbreak detection and investigation. Clin Infect Dis.

[78] Dallman TJ, Byrne L, Ashton PM, Cowley LA, Perry NT, Adak G (2015). Whole-genome sequencing for national surveillance of Shiga toxin-producing *Escherichia coli* O157. Clin Infect Dis.

[79] Holmes A, Allison L, Ward M, Dallman TJ, Clark R, Fawkes A (2015). Utility of whole-genome sequencing of *Escherichia coli* O157 for outbreak detection and epidemiological surveillance. J Clin Microbiol.

[80] Chattaway MA, Dallman TJ, Gentle A, Wright MJ, Long SE, Ashton PM, et al. Whole genome sequencing for public health surveillance of Shiga Toxin-producing *Escherichia coli* other than serogroup o157. Front Microbiol. 2016. 10.3389/fmicb.2016.00258. 10.3389/fmicb.2016.00258 PMC4776118 26973632

[81] Ford Laura, Carter Glen P., Wang Qinning, Seemann Torsten, Sintchenko Vitali, Glass Kathryn, Williamson Deborah A., Howard Peter, Valcanis Mary, Castillo Cristina Fabiola Sotomayor, Sait Michelle, Howden Benjamin P., Kirk Martyn D. (2018). Incorporating Whole-Genome Sequencing into Public Health Surveillance: Lessons from Prospective Sequencing of Salmonella Typhimurium in Australia. Foodborne Pathogens and Disease.

[82] Chattaway MA, Greig DR, Gentle A, Hartman HB, Dallman TJ, Jenkins C. Whole-genome sequencing for national surveillance of *Shigella flexneri*. Front Microbiol. 2017;8(1700). 10.3389/fmicb.2017.01700. 10.3389/fmicb.2017.01700 PMC5610704 28974944

[83] Gymoese P, Sørensen G, Litrup E, Olsen JE, Nielsen EM, Torpdahl M (2017). Investigation of outbreaks of *Salmonella enterica* serovar typhimurium and its monophasic variants using whole-genome sequencing Denmark. Emerg Infect Dis.

[84] Dallman TJ, Chattaway MA, Mook P, Godbole G, Crook PD, Jenkins C (2016). Use of whole genome sequencing for the public health surveillance of *Shigella sonnei* in England and Wales, 2015. J Med Microbiol.

[85] Aanensen DM, Feil EJ, Holden MTG, Dordel J, Yeats CA, Fedosejev A (2016). Whole-genome sequencing for routine pathogen surveillance in public health: a population snapshot of invasive *Staphylococcus aureus* in Europe. MBio.

[86] Sonda T, Kumburu H, van Zwetselaar M, Alifrangis M, Mmbaga BT, Lund O (2018). Molecular epidemiology of virulence and antimicrobial resistance determinants in *Klebsiella pneumoniae* from hospitalised patients in Kilimanjaro, Tanzania. Eur J Clin Microbiol Infect Dis.

[87] Jajou R, De Neeling A, Van Hunen R, De Vries G, Schimmel H, Mulder A (2018). Epidemiological links between tuberculosis cases identified twice as efficiently by whole genome sequencing than conventional molecular typing: a population-based study. PLoS One.

[88] Brown TS, Narechania A, Walker JR, Planet PJ, Bifani PJ, Kolokotronis SO, et al. Genomic epidemiology of Lineage 4 *Mycobacterium tuberculosis* subpopulations in New York City and New Jersey, 1999–2009. BMC Genomics. 2016. 10.1186/s12864-016-3298-6. 10.1186/s12864-016-3298-6 PMC5117616 27871225

[89] Stucki D, Ballif M, Egger M, Furrer H, Altpeter E, Battegay M (2016). Standard genotyping overestimates transmission of *Mycobacterium tuberculosis* among immigrants in a low incidence country. J Clin Microbiol.

[90] Gurjav U, Outhred AC, Jelfs P, McCallum N, Wang Q, Hill-Cawthorne GA (2016). Whole genome sequencing demonstrates limited transmission within identified Mycobacterium tuberculosis clusters in New South Wales. Australia. PLoS One.

[91] Dyson ZA, Thanh DP, Bodhidatta L, Mason CJ, Srijan A, Rabaa MA (2017). Whole genome sequence analysis of *Salmonella* Typhi isolated in Thailand before and after the introduction of a national immunization program. PLoS Negl Trop Dis.

[92] Hill DMC, Lucidarme J, Gray SJ, Newbold LS, Ure R, Brehony C (2015). Genomic epidemiology of age-associated meningococcal lineages in national surveillance: an observational cohort study. Lancet Infect Dis.

[93] Moore CE, Giess A, Soeng S, Sar P, Kumar V, Nhoung P (2016). Characterisation of invasive *Streptococcus pneumoniae* isolated from Cambodian children between 2007-2012. PLoS One.

[94] Duvvuri VR, Deng X, Teatero S, Memari N, Athey T, Fittipaldi N (2016). Population structure and drug resistance patterns of emerging non-PCV-13 *Streptococcus pneumoniae* serotypes 22F, 15A, and 8 isolated from adults in Ontario. Canada. Infect Genet Evol.

[95] Sealey KL, Harris SR, Fry NK, Hurst LD, Gorringe AR, Parkhill J (2015). Genomic analysis of isolates from the United Kingdom 2012 pertussis outbreak reveals that vaccine antigen genes are unusually fast evolving. J Infect Dis.

[96] Xu Y, Liu B, Gröndahl-Yli-Hannuksila K, Tan Y, Feng L, Kallonen T, et al. Whole-genome sequencing reveals the effect of vaccination on the evolution of Bordetella pertussis. Sci Rep. 2015;5(12888). Available from: 10.1038/srep12888. 10.1038/srep12888 PMC4539551 26283022

[97] Mowlaboccus S, Perkins TT, Smith H, Sloots T, Tozer S, Prempeh LJ (2016). Temporal changes in BEXSERO® antigen sequence type associated with genetic lineages of *Neisseria meningitidis* over a 15-year period in Western Australia. PLoS One.

[98] Rodrigues CMC, Lucidarme J, Borrow R, Smith A, Cameron JC, Moxon ER (2018). Genomic surveillance of 4CMenB vaccine antigenic variants among disease-causing *Neisseria meningitidis* isolates, United Kingdom, 2010-2016. Emerg Infect Dis.

[99] Sidikou F, Zaneidou M, Alkassoum I, Schwartz S, Issaka B, Obama R (2016). Emergence of epidemic *Neisseria meningitidis* serogroup C in Niger, 2015: an analysis of national surveillance data. Lancet Infect Dis.

[100] Chochua S, Metcalf BJ, Li Z, Walker H, Tran T, McGee L (2017). Invasive serotype 35B pneumococci including an expanding serotype switch lineage, United States, 2015–2016. Emerg Infect Dis.

[101] Mowlaboccus S, Mullally CA, Richmond PC, Howden BP, Stevens K, Speers DJ (2017). Differences in the population structure of *Neisseria meningitidis* in two Australian states: Victoria and Western Australia. PLoS One.

[102] Ellington MJ, Reuter S, Harris SR, Holden MTG, Cartwright EJ, Greaves D (2015). Emergent and evolving antimicrobial resistance cassettes in community-associated fusidic acid and meticillin-resistant *Staphylococcus aureus*. Int J Antimicrob Agents.

[103] Hughes J, Stabler R, Gaunt M, Karadag T, Desai N, Betley J, et al. Clonal variation in high- and low-level phenotypic and genotypic mupirocin resistance of MRSA isolates in south-east London. J Antimicrob Chemother. 2015. 10.1093/jac/dkv248. 10.1093/jac/dkv248 26316381

[104] Alam MT, Read TD, Petit RA, Boyle-Vavra S, Miller LG, Eells SJ (2015). Transmission and microevolution of USA300 MRSA in U.S. households: evidence from whole-genome sequencing. MBio.

[105] Chow A, Lim VW, Khan A, Pettigrew K, Lye DCB, Kanagasabai K (2017). MRSA transmission dynamics among interconnected acute, intermediate-term, and long-term healthcare facilities in Singapore. Clin Infect Dis.

[106] Harrison EM, Ludden C, Brodrick HJ, Blane B, Brennan G, Morris D, et al. Transmission of methicillin-resistant *Staphylococcus aureus* in long-term care facilities and their related healthcare networks. Genome Med. 2016. 10.1186/s13073-016-0353-5. 10.1186/s13073-016-0353-5 PMC5048656 27716432

[107] Bowen AC, Harris T, Holt DC, Giffard PM, Carapetis JR, Campbell PT (2016). Whole genome sequencing reveals extensive community-level transmission of group A *Streptococcus* in remote communities. Epidemiol Infect.

[108] Widmer AF, Frei R, Erb S, Stranden A, Kuijper EJ, Knetsch CW, et al. Transmissibility of *Clostridium difficile* without contact isolation: results from a prospective observational study with 451 patients. Clin Infect Dis. 2017. 10.1093/cid/ciw758.. 10.1093/cid/ciw758 28172613

[109] Guerra-Assunção JA, Crampin AC, Houben RMGJ, Mzembe T, Mallard K, Coll F (2015). Large-scale whole genome sequencing of M. tuberculosis provides insights into transmission in a high prevalence area. Elife.

[110] Bosch T, Witteveen S, Haenen A, Landman F, Schouls LM (2016). Next generation sequencing confirms presumed nosocomial transmission of LA-MRSA in the Netherlands. Appl Environ Microbiol.

[111] Earls MR, Kinnevey PM, Brennan GI, Lazaris A, Skally M, O’Connell B (2017). The recent emergence in hospitals of multidrug-resistant community-associated sequence type 1 and spa type t127 methicillin-resistant *Staphylococcus aureus* investigated by whole-genome sequencing: Implications for screening. PLoS One.

[112] Price JR, Cole K, Bexley A, Kostiou V, Eyre DW, Golubchik T (2017). Transmission of *Staphylococcus aureus* between health-care workers, the environment, and patients in an intensive care unit: a longitudinal cohort study based on whole-genome sequencing. Lancet Infect Dis.

[113] Arnott A, Wang Q, Bachmann N, Sadsad R, Biswas C, Sotomayor C (2018). Multidrug-resistant *Salmonella enterica* 4,[5],12:i:- Sequence Type 34, New South Wales, Australia, 2016–2017. Emerg Infect Dis.

[114] Moradigaravand D, Martin V, Peacock SJ, Parkhill J (2017). Evolution and epidemiology of multidrug-resistant *Klebsiella pneumoniae* in the United Kingdom and Ireland. MBio.

[115] Hargreaves ML, Shaw KM, Dobbins G, Snippes Vagnone PM, Harper JE, Boxrud D (2015). Clonal Dissemination of *Enterobacter cloacae* Harboring *blaKPC-3* in the Upper Midwestern United States. Antimicrob Agents Chemother.

[116] Steglich M, Nitsche A, Von Müller L, Herrmann M, Kohl TA, Niemann S (2015). Tracing the spread of clostridium difficile ribotype 027 in Germany based on bacterial genome sequences. PLoS One.

[117] Pecora ND, Li N, Allard M, Li C, Albano E, Delaney M (2015). Genomically informed surveillance for carbapenem-resistant Enterobacteriaceae in a health care system. MBio.

[118] Peirano Gisele, Matsumura Yasufumi, Adams Mark D., Bradford Patricia, Motyl Mary, Chen Liang, Kreiswirth Barry N., Pitout Johann D.D. (2018). Genomic Epidemiology of Global Carbapenemase-Producing Enterobacter spp., 2008–2014. Emerging Infectious Diseases.

[119] Schaufler Katharina, Semmler Torsten, Wieler Lothar H., Wöhrmann Michael, Baddam Ramani, Ahmed Niyaz, Müller Kerstin, Kola Axel, Fruth Angelika, Ewers Christa, Guenther Sebastian (2015). Clonal spread and interspecies transmission of clinically relevant ESBL-producingEscherichia coliof ST410—another successful pandemic clone?. FEMS Microbiology Ecology.

[120] Sandoval-Azuara SE, Muñiz-Salazar R, Perea-Jacobo R, Robbe-Austerman S, Perera-Ortiz A, López-Valencia G (2017). Whole genome sequencing of *Mycobacterium bovis* to obtain molecular fingerprints in human and cattle isolates from Baja California. Mexico. Int J Infect Dis.

[121] Edirmanasinghe R, Finley R, Parmley EJ, Avery BP, Carson C, Bekal S (2017). A whole-genome sequencing approach to study cefoxitin-resistant *Salmonella enterica* serovar Heidelberg isolates from various sources. Antimicrob Agents Chemother.

[122] Falgenhauer L, Imirzalioglu C, Ghosh H, Gwozdzinski K, Schmiedel J, Gentil K (2016). Circulation of clonal populations of fluoroquinolone-resistant CTX-M-15-producing *Escherichia coli* ST410 in humans and animals in Germany. Int J Antimicrob Agents.

[123] Fischer J, Hille K, Ruddat I, Mellmann A, Köck R, Kreienbrock L (2017). Simultaneous occurrence of MRSA and ESBL-producing Enterobacteriaceae on pig farms and in nasal and stool samples from farmers. Vet Microbiol.

[124] Grøntvedt CA, Elstrøm P, Stegger M, Skov RL, Andersen PS, Larssen KW (2016). Methicillin-resistant *Staphylococcus aureus* CC398 in humans and pigs in Norway: a “One Health” perspective on introduction and transmission. Clin Infect Dis.

[125] Sparham SJ, Kwong JC, Valcanis M, Easton M, Trott DJ, Seemann T (2017). Emergence of multidrug resistance in locally-acquired human infections with *Salmonella* Typhimurium in Australia owing to a new clade harbouring *bla*CTX-M-9. Int J Antimicrob Agents.

[126] Tate H, Folster JP, Hsu C-H, Chen J, Hoffmann M, Li C, et al. Comparative analysis of extended-spectrum-B-lactamase CTX-M-65-producing *Salmonella enterica* serovar Infantis isolates from humans, food animals, and retail chickens in the United States. Antimicrob Agents Chemother. 2017;61(7). 10.1128/AAC.00488-17. 10.1128/AAC.00488-17 PMC5487606 28483962

[127] Chand M, Lamagni T, Kranzer K, Hedge J, Moore G, Parks S (2017). Insidious risk of severe *Mycobacterium chimaera* infection in cardiac surgery patients. Clin Infect Dis.

[128] Afset J, Larssen K, Bergh K, Lärkeryd A, Sjödin A, Johansson A, Forsman M (2015). Phylogeographical pattern of Francisella tularensis in a nationwide outbreak of tularaemia in Norway, 2011. Eurosurveillance.

[129] Burall LS, Grim CJ, Datta AR (2017). A clade of *Listeria monocytogenes* serotype 4b variant strains linked to recent listeriosis outbreaks associated with produce from a defined geographic region in the US. PLoS One.

[130] Dangel A, Berger A, Konrad R, Bischoff H, Sing A (2018). Geographically diverse clusters of nontoxigenic *Corynebacterium diphtheriae* infection, Germany, 2016–2017. Emerg Infect Dis.

[131] Deng X, Peirano G, Schillberg E, Mazzulli T, Gray-Owen SD, Wylie JL (2016). Whole-genome sequencing reveals the origin and rapid evolution of an emerging outbreak strain of *Streptococcus pneumoniae* 12F. Clin Infect Dis.

[132] Layer F, Sanchini A, Strommenger B, Cuny C, Breier AC, Proquitté H (2015). Molecular typing of toxic shock syndrome toxin-1- and Enterotoxin A-producing methicillin-sensitive *Staphylococcus aureus* isolates from an outbreak in a neonatal intensive care unit. Int J Med Microbiol.

[133] Rimoldi SG, Gentile B, Pagani C, Di Gregorio A, Anselmo A, Palozzi AM, et al. Whole genome sequencing for the molecular characterization of carbapenem-resistant *Klebsiella pneumoniae* strains isolated at the Italian ASST Fatebenefratelli Sacco Hospital, 2012-2014. BMC Infect Dis. 2017. 10.1186/s12879-017-2760-7. 10.1186/s12879-017-2760-7 PMC5634883 29017452

[134] Ferdous M, Friedrich AW, Grundmann H, de Boer RF, Croughs PD, Islam MA (2016). Molecular characterization and phylogeny of Shiga toxin–producing *Escherichia coli* isolates obtained from two Dutch regions using whole genome sequencing. Clin Microbiol Infect.

[135] Toleman MS, Reuter S, Coll F, Harrison EM, Blane B, Brown NM (2016). Systematic surveillance detects multiple silent introductions and household transmission of methicillin-resistant *Staphylococcus aureus* USA300 in the East of England. J Infect Dis.

[136] Waldram A, Dolan G, Ashton PM, Jenkins C, Dallman TJ (2018). Epidemiological analysis of *Salmonella* clusters identified by whole genome sequencing, England and Wales 2014. Food Microbiol.

[137] Demczuk W, Lynch T, Martin I, Van Domselaar G, Graham M, Bharat A (2015). Whole-genome phylogenomic heterogeneity of *Neisseria gonorrhoeae* isolates with decreased cephalosporin susceptibility collected in Canada between 1989 and 2013. J Clin Microbiol.

[138] Gray MD, Lacher DW, Leonard SR, Abbott J, Zhao S, Lampel KA (2015). Prevalence of Stx-producing *Shigella* species isolated from French travelers returning from the Caribbean: an emerging pathogen with international implications. Clin Microbiol Infect.

[139] Pham Thanh D, Thompson CN, Rabaa MA, Sona S, Sopheary S, Kumar V (2016). The molecular and spatial epidemiology of typhoid fever in rural Cambodia. PLoS Negl Trop Dis.

[140] Sonda T, Kumburu H, van Zwetselaar M, Alifrangis M, Mmbaga BT, Lund O (2018). Prevalence and risk factors for CTX-M gram-negative bacteria in hospitalized patients at a tertiary care hospital in Kilimanjaro, Tanzania. Eur J Clin Microbiol Infect Dis.

[141] Chalker V, Jironkin A, Coelho J, Al-Shahib A, Platt S, Kapatai G, et al. Genome analysis following a national increase in Scarlet Fever in England 2014. BMC Genomics. 2017. 10.1186/s12864-017-3603-z. 10.1186/s12864-017-3603-z PMC5345146 28283023

[142] Brodrick HJ, Raven KE, Harrison EM, Blane B, Reuter S, Török ME, et al. Whole-genome sequencing reveals transmission of vancomycin-resistant *Enterococcus faecium* in a healthcare network. Genome Med. 2016. 10.1186/s13073-015-0259-7. 10.1186/s13073-015-0259-7 PMC4709893 26759031

[143] Stoesser N, Xayaheuang S, Vongsouvath M, Phommasone K, Elliott I, Del Ojo EC, et al. Colonization with Enterobacteriaceae producing ESBLs in children attending pre-school childcare facilities in the Lao People’s Democratic Republic. J Antimicrob Chemother. 2015. 10.1093/jac/dkv021. 10.1093/jac/dkv021 PMC4498295 25681128

[144] Coscolla M, Barry PM, Oeltmann JE, Koshinsky H, Shaw T, Cilnis M (2015). Genomic epidemiology of multidrug-resistant *Mycobacterium tuberculosis* during transcontinental spread. J Infect Dis.

[145] Lee RS, Radomski N, Proulx J, Manry J, McIntosh F, Desjardins F (2015). Re-emergence and amplification of tuberculosis in the Canadian Arctic. J Infect Dis.

[146] Halbedel S, Prager R, Fuchs S, Trost E, Werner G, Flieger A. Whole-genome sequencing of recent *Listeria monocytogenes* isolates from Germany reveals population structure and disease clusters. J Clin Microbiol. 2018, 56;(6). 10.1128/JCM.00119-18. 10.1128/JCM.00119-18 PMC5971532 29643197

[147] Kvistholm Jensen A, Møller Nielsen E, Torgny Björkman J, Jensen T, Müller L, Persson S (2016). Whole-genome sequencing used to investigate a nationwide outbreak of listeriosis caused by ready-to-eat delicatessen meat, Denmark, 2014. Clin Infect Dis.

[148] Grant K, Jenkins C, Arnold C, Green J, Zambon M. Implementing pathogen genomics: a case study. 2018. Available from: https://assets.publishing.service.gov.uk/government/uploads/system/uploads/attachment_data/file/731057/implementing_pathogen_genomics_a_case_study.pdf

[149] Bartels M, Larner-Svensson H, Meiniche H, Kristoffersen K, Schønning K, Nielsen J, Rohde S, Christensen L, Skibsted A, Jarløv J, Johansen H, Andersen L, Petersen I, Crook D, Bowden R, Boye K, Worning P, Westh H (2015). Monitoring meticillin resistant Staphylococcus aureus and its spread in Copenhagen, Denmark, 2013, through routine whole genome sequencing. Eurosurveillance.

[150] Pinholt M, Larner-Svensson H, Littauer P, Moser CE, Pedersen M, Lemming LE (2015). Multiple hospital outbreaks of vanA *Enterococcus faecium* in Denmark, 2012-13, investigated by WGS. MLST and PFGE. J Antimicrob Chemother.

[151] European Centre for Disease Prevention and Control. Monitoring the use of whole-genome sequencing in infectious disease surveillance in Europe 2015–2017. 2017. Available from: https://ecdc.europa.eu/sites/portal/files/documents/monitoring-WGS-infectious-disease-surveillance-in-Europe-2015-2017-updated-Dec-2018.pdf.

[152] Mellmann A, Bletz S, Böking T, Kipp F, Becker K, Schultes A (2016). Real-time genome sequencing of resistant bacteria provides precision infection control in an institutional setting. J Clin Microbiol.

[153] Mulhall RM, Brehony C, O’Connor L, Meyler K, Jolley KA, Bray J (2016). Resolution of a protracted Serogroup B meningococcal outbreak with whole-genome sequencing shows interspecies genetic transfer. J Clin Microbiol.

[154] Schlebusch S, Price GR, Gallagher RL, Horton-Szar V, Elbourne LDH, Griffin P (2017). MALDI-TOF MS meets WGS in a VRE outbreak investigation. Eur J Clin Microbiol Infect Dis.

[155] Mossong J, Decruyenaere F, Moris G, Ragimbeau C, Olinger CM, Johler S, et al. Investigation of a staphylococcal food poisoning outbreak combining case-control, traditional typing and whole genome sequencing methods, Luxembourg, June 2014. Eurosurveillance. 2015;20(45):pii = 30059. Available from: 10.2807/1560-7917.ES.2015.20.45.30059 10.2807/1560-7917.ES.2015.20.45.30059 26608881

[156] Weterings V, Zhou K, Rossen JW, van Stenis D, Thewessen E, Kluytmans J (2015). An outbreak of colistin-resistant *Klebsiella pneumoniae* carbapenemase-producing Klebsiella pneumoniae in the Netherlands (July to December 2013), with inter-institutional spread. Eur J Clin Microbiol Infect Dis.

[157] Didelot X, Bowden R, Wilson DJ, Peto TEA, Crook DW (2012). Transforming clinical microbiology with bacterial genome sequencing. Nat Rev Genet.

[158] Rowell S, King C, Jenkins C, Dallman TJ, Decraene V, Lamden K (2016). An outbreak of Shiga toxin-producing *Escherichia coli* serogroup O157 linked to a lamb-feeding event. Epidemiol Infect.

[159] Pightling AW, Pettengill JB, Luo Y, Baugher JD, Rand H, Strain E. Interpreting whole-genome sequence analyses of foodborne bacteria for regulatory applications and outbreak investigations. Front Microbiol. 2018. 10.3389/fmicb.2018.01482. 10.3389/fmicb.2018.01482 PMC6048267 30042741

[160] Grad YH, Lipsitch M. Epidemiologic data and pathogen genome sequences: a powerful synergy for public health. Genome Biol. 2014. 10.1186/s13059-014-0538-4. 10.1186/s13059-014-0538-4 PMC4282151 25418119

[161] Fitzpatrick MA, Ozer EA, Hauser AR (2016). Utility of whole-genome sequencing in characterizing *Acinetobacter* epidemiology and analyzing hospital outbreaks. J Clin Microbiol.

[162] Tyler AD, Randell E, Baikie M, Antonation K, Janella D, Christianson S (2017). Application of whole genome sequence analysis to the study of *Mycobacterium tuberculosis* in Nunavut Canada. PLoS One.

[163] Muellner P, Stark KDC, Dufour S, Zadoks RN (2016). ‘Next- Generation’ surveillance: an epidemiologists’ perspective on the use of molecular information in food safety and animal health decision-making. Zoonoses Public Health.

[164] Franz E, Gras LM, Dallman T (2016). Significance of whole genome sequencing for surveillance, source attribution and microbial risk assessment of foodborne pathogens. Curr Opin Food Sci.

[165] Hill AA, Crotta M, Wall B, Good L, O’Brien SJ, Guitian J (2017). Towards an integrated food safety surveillance system: a simulation study to explore the potential of combining genomic and epidemiological metadata. R Soc Open Sci.

[166] Tagini F, Aubert B, Troillet N, Pillonel T, Praz G, Crisinel PA (2017). Importance of whole genome sequencing for the assessment of outbreaks in diagnostic laboratories: analysis of a case series of invasive *Streptococcus pyogenes* infections. Eur J Clin Microbiol Infect Dis.

[167] Neuert S, Nair S, Day MR, Doumith M, Ashton PM, Mellor KC, et al. Prediction of phenotypic antimicrobial resistance profiles from whole genome sequences of non-typhoidal *Salmonella enterica*. Front Microbiol. 2018. 10.3389/fmicb.2018.00592. 10.3389/fmicb.2018.00592 PMC5880904 29636749

[168] Salathé M. Digital epidemiology: what is it, and where is it going? Life Sci Soc Policy. 2018;14(1). 10.1186/s40504-017-0065-7. 10.1186/s40504-017-0065-7 PMC5754279 29302758

[169] Johnson I, Hansen A, Bi P (2018). The challenges of implementing an integrated One Health surveillance system in Australia. Zoonoses Public Health.

[170] Rantsiou Kalliopi, Kathariou Sophia, Winkler Annet, Skandamis Panos, Saint-Cyr Manuel Jimmy, Rouzeau-Szynalski Katia, Amézquita Alejandro (2018). Next generation microbiological risk assessment: opportunities of whole genome sequencing (WGS) for foodborne pathogen surveillance, source tracking and risk assessment. International Journal of Food Microbiology.

[171] Kao RR, Haydon DT, Lycett SJ, Murcia PR (2014). Supersize me: how whole-genome sequencing and big data are transforming epidemiology. Trends Microbiol.

[172] Luheshi L, Raza S, Moorthie S, Hall A, Blackburn L, Rands C (2015). Pathogen genomics into practice.

