# Supplementary material for: Status and potential of bacterial genomics for public health practice: a scoping review
Source: Implement Sci. 2019 Aug 13;14:79. doi: 10.1186/s13012-019-0930-2 (PMC6692930; doi:10.1186/s13012-019-0930-2)
Supplement: Supplementary file 2 — Protocol. (PDF 939 kb) [file 13012_2019_930_MOESM2_ESM.pdf]

STATUS AND POTENTIAL OF BACTERIAL GENOMICS FOR PUBLIC HEALTH PRACTICE: A SCOPING REVIEW

BACKGROUND

The development of sequencing technologies, especially the shift from traditional Sanger sequencing to next-generation sequencing (NGS), has provided new opportunities for applications in various fields, including infectious disease epidemiology (1). NGS offers new opportunities in research, individual patient care, and public health. However, this review only focuses on the **public health** aspects (i.e. application of NGS for population-level management of infectious diseases). This review will not discuss technical aspects of the sequence technologies, the use of NGS for individual patient care, nor the use of NGS for research purposes (see Figure 1 and 2).

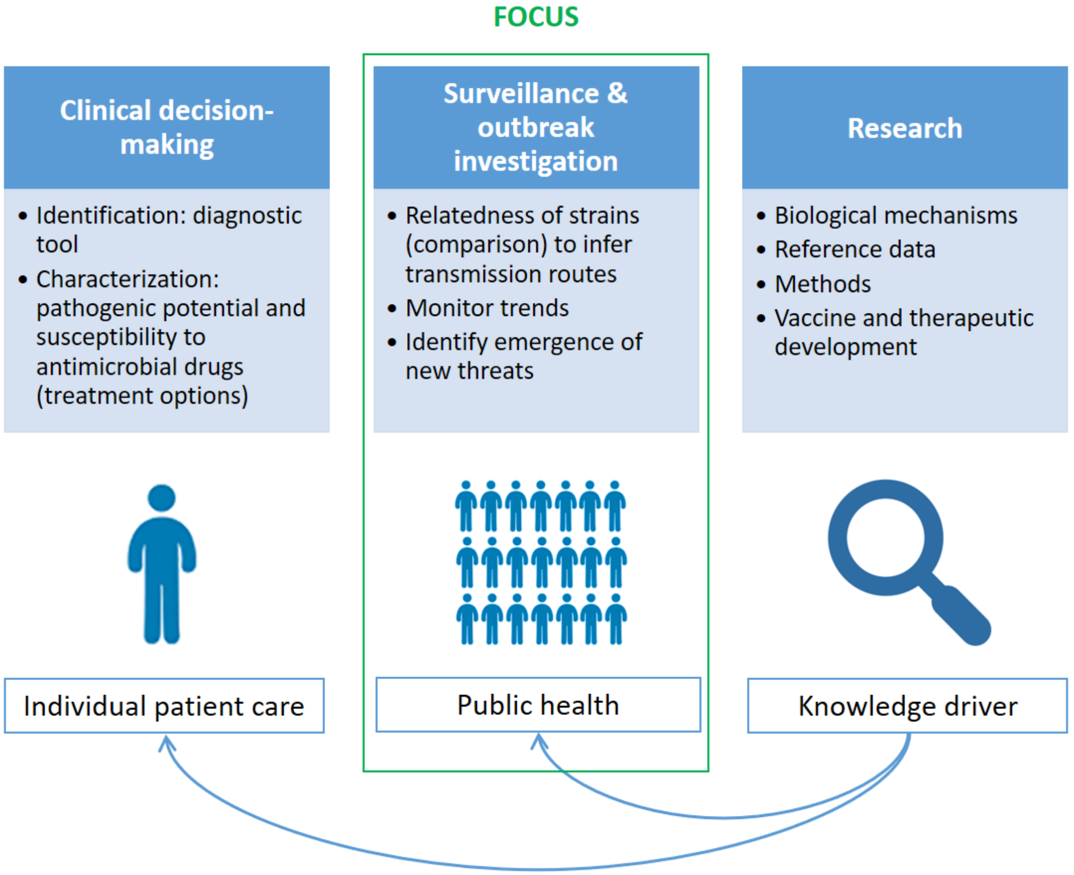

Figure 1. Applications of NGS. Three domains are considered as users of next-generation sequencing (NGS) technologies. NGS has the ability to inform and improve individual patient care, by identifying the species, determining its pathogenic potential and testing its susceptibility to antimicrobial drugs. NGS also provides data for public health surveillance about the relatedness of the pathogen to other strains to investigate transmission routes, monitor trends and to allow the identification of outbreaks and new threats. Research is a knowledge driver providing reference data, methods, and a deeper understanding about the underlying mechanisms to the other domains. Here, we focus on the public health perspective, i.e. on the level of the population.

Developments in sequencing technologies are affecting the monitoring and control of all pathogens, including viruses, bacteria, fungi and parasites. This review focuses on **bacterial pathogens** to demonstrate the likely changes from an **epidemiologist's perspective** that arise from the implementation of routine whole-genome sequencing.

As NGS is a fast-evolving technique, and as our main interest are its state-of-the-art applications, we restrict this review to recent publications, i.e. studies published between **2015** and 2018.

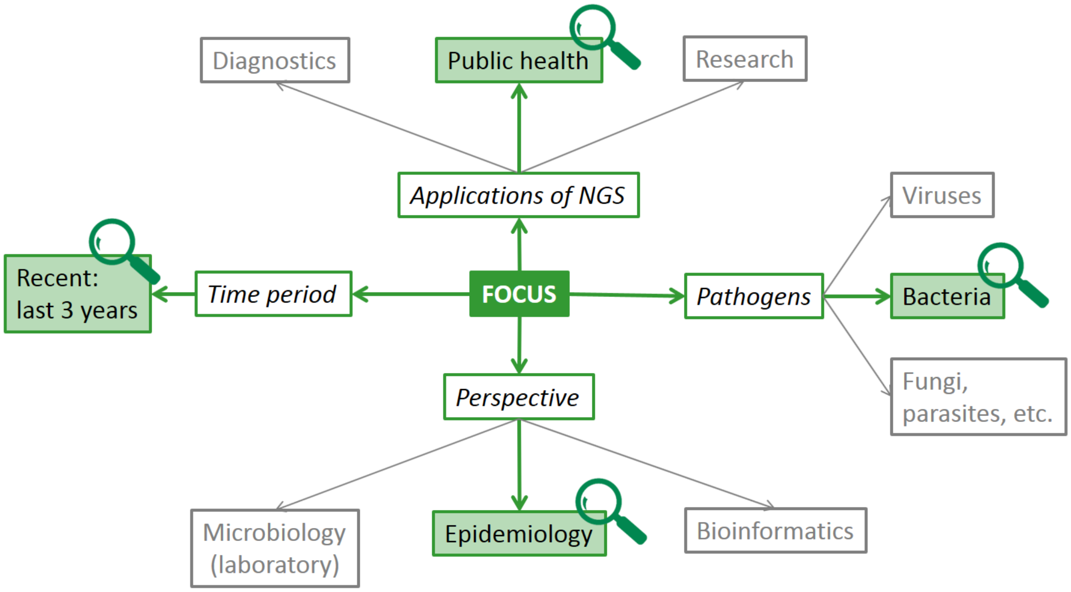

Figure 2. Focus of the scoping review. Focus of this scoping review are the applications of NGS on bacterial pathogens for public health practice recently published, from an epidemiologist's perspective.

RESEARCH QUESTION

What is the current status and the potential of next-generation sequencing (NGS) for routine public health practices related to surveillance and control of bacterial infections?

- What are the **applications** of NGS for the surveillance and control of bacterial infections?
- What is the **added value** of implementing NGS for routine public health practices related to the surveillance and control of bacterial infections?
- What are the **challenges** related to the implementation of NGS for routine public health practices related to the surveillance and control of bacterial infections?

**Population**	Humans infected with a bacteria
**Concept**	Use of NGS on the bacterial isolates (pathogen genomics): status and potential<br>- Applications<br>- Added value<br>- Challenges
**Context**	Public health practice (outbreak investigations and surveillance)

PURPOSE AND OBJECTIVES

Collective experience on the use of next-generation sequencing (NGS) for routine public health practices related to communicable diseases is spread across literature. This scoping review aims to summarize the findings of studies that applied NGS for public health practices, i.e. in outbreak investigations and surveillance. The first objective is to identify and describe the **applications** of NGS in the context of routine public health related to communicable diseases across the globe. The secondary objectives are to describe the reported **added value** of NGS compared to traditional methods, and the **challenges** and needs related to the implementation of NGS in routine public health practice. We provide an epidemiologists' perspective on the use of NGS in public health surveillance and control of communicable diseases to complement previous reviews that focus on technical, diagnostic or pathogen specific aspects. The purpose of this scoping review is to summarize the experience gained and use it to further advance the implementation of NGS in routine public health.

A preliminary search of the literature was conducted in the following databases: JBI Database of Scoping Reviews and Implementation Reports, PROSPERO International Prospective Register of Systematic Reviews, and Epistemonikos which confirmed that no systematic or scoping review has been published or is currently underway on this topic.

METHODS

Scoping reviews are intended to provide an overview of the nature and extent of the literature on a topic via systematically searching, selecting and summarizing evidence. A scoping review can be of particular use when the topic is of a complex or heterogeneous nature (2), which is appropriate for public health activities related to the prevention and control of infectious diseases. This review will be conducted according to the Preferred Reporting Items for Systematic Reviews and Meta-Analyses (PRISMA) guidelines, adapted for use in a scoping review as appropriate, and adhering to the methodology outlined in The Joanna Briggs Institute Manual for Scoping reviews. In addition, the framework outlined by Arksey and O'Malley (2005) (3) in their methodological paper on scoping reviews will be followed, as well as the methodology of recent publications (4,5).

The main steps of this scoping review are to (1) conduct a systematic search of the published literature applying pathogen genomics for public health practice; (2) map the characteristics of the identified studies; (3) map the range of applications identified, and (5) describe the reported added value, challenges and needs related to the implementation of NGS from an epidemiologists' perspective.

CRITERIA FOR CONSIDERING STUDIES

i. <u>Types of studies</u>

- Prospective cohort studies
- Retrospective cohort studies
- Cross-sectional studies
- Case-control studies

- Case series

ii. Participants/materials

- Individuals infected with an infectious disease caused by a bacteria.
- Isolates/strains extracted from samples from above individuals.

iii. Setting

- Community
- Institutional (hospital, nursing home, etc.)

iv. Eligibility criteria

A scoping review has a broader ‘scope’ with correspondingly less restrictive inclusion criteria. The inclusion criteria are organized following the PCC (Population, Concept and Context) elements. Screening the articles and applying the inclusion criteria will be done using an iterative and flexible approach. Studies must include at least 2 individuals with a bacterial infection and NGS must be applied on the bacterial isolates. Studies must describe the application of NGS from a public health perspective (i.e. population-level). Therefore, the main study aims should be within the context of an outbreak investigation, control-oriented surveillance, or strategy-oriented surveillance. Studies focusing on technical aspects, applying NGS solely for individual patient care, and using NGS for research purposes will be excluded. Further, only studies applying NGS within a real-life public health setting, as opposed to an experimental setting, and producing an output that can be directly translated into actionable results to benefit public health, will be included. This also includes proof-of-concept studies mimicking real-life public health problems and of which the results can be used to advance future implementation of NGS. Studies published between January 2015 and February 2018 (with an updated search in September 2018) were included to consider the most current activities in this fast-evolving field.

Inclusion	Exclusion
Language: English full text<br>Timeframe: studies published between 1/1/2015 and 4/9/2018<br>All geographical regions<br>Studies with primary NGS data available or describing the implementation of NGS	No English full text available<br>Studies published before 1/1/2015<br>/<br>Conference abstracts or literature reviews
Population	
Studies must include at least two individuals with a bacterial infection.	Non-human studies, i.e. studies only including environmental or animal samples.
Concept	
Studies must apply NGS on the bacterial isolates (pathogen genomics), more specifically studies with a strong focus on the use of NGS (as a primary typing tool or being a determining factor for the outcome of the study)	No use of NGS (e.g. microarray studies).<br>Use of NGS for a small, insignificant subset of samples, i.e. studies where NGS does not play a major role and/or where there is not focus on the use/application/added value/challenges of NGS.<br>Not applying NGS on bacterial isolates (e.g. studies only sequencing host genomes, studies only sequencing pathogens other than bacteria).
Context	
To be considered genomic epidemiology, study aims should include at least one of the following:<br>- Outbreak investigation (source tracing, interrupt transmission, feedback on key phenotypic attributes)<br>- Control-oriented surveillance (early outbreak detection, identify the emergence of new threats, understand transmission dynamics)<br>- Strategy-oriented surveillance (overview of circulating strains, understand transmission dynamics, evaluation of control programs, identification of risk factors)	Studies that do not focus on outbreak investigations or surveillance and control (i.e. not for public health practice), for example:<br>- Studies only focusing on technical aspects of the sequence technologies (e.g. benchmarking of sequencing methods, comparison of sequencing methods, description of sequencing methods, etc.)<br>- Studies only/mainly focusing on bioinformatics aspects (e.g. developing new typing schemes)<br>- Studies using NGS for research purposes (e.g. underlying biological mechanisms, cellular processes, evolutionary aspects, etc.)<br>- Studies using NGS to identify biological markers that can be used for future routine public health practices
Studies must describe the application of NGS at the population level (i.e. from a public health perspective)	Studies not focusing on the population perspective:<br>- Studies using NGS solely for individual patient care (e.g. diagnostic studies - studies examining whole genome sequencing for prediction of phenotypic drug resistance or studies that exclusively test new diagnostic approaches without meeting the above inclusion criteria)<br>- Studies including less than two clinical samples from patients, apart from environmental or animal samples.<br>- Studies describing individual patients/cases (i.e. case studies)
Studies using NGS within a real-life public health setting producing an output that can be directly translated into actionable results for public health (including proof-of-concept studies).	Studies using NGS in an experimental setting (e.g. using a small convenience sample of historic isolates, only suggesting the use of NGS in a routine public health setting but not yet applying it, etc.)

Decision tree for the inclusion of studies in this review based on the context (green = include)

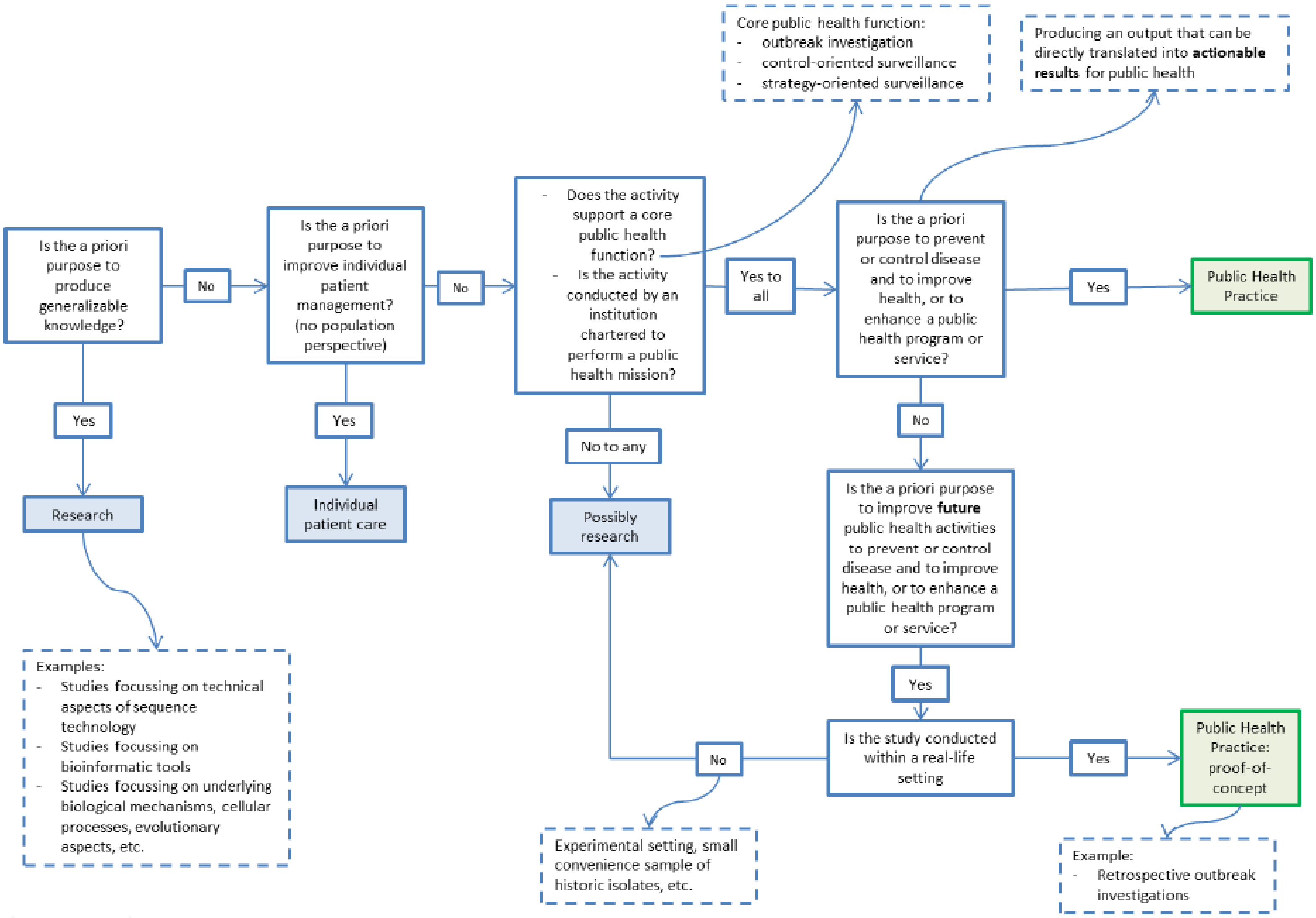

TYPES OF OUTCOME MEASURES

- Description of the different **applications** of NGS for public health related to infectious diseases.
- Description of the **added value** of NGS for outbreak investigations/routine surveillance (pathogen and application specific).
- Description of the **challenges** related to the implementation of NGS in routine public health surveillance/outbreak investigations of infectious diseases.

SEARCH METHODS

The approach to search for studies during a scoping review follows the same three-step method recommended as in standard JBI systematic reviews. An initial search of Medline was undertaken, followed by an analysis of the text words contained in the title, abstract and index terms used to describe the articles. This informed the development of the search strategy, including identified keywords and index terms. A comprehensive search using all the identified keywords and index terms will be undertaken. Lastly, the reference list of all articles subject to full text review will be screened for additional studies and assessed for suitability based on the studies title and abstract.
The search will be limited to studies published in English in the last 3 years (2015–2018). This period is chosen to only address recent publications within this fast-evolving field.

i. Systematic electronic search

A compound search strategy will be developed to identify all relevant studies. The MEDLINE bibliographic database was searched using the PubMed search engine.

ii. Other resources (*ad hoc* search)

Reference lists of all studies identified by the above methods and bibliographies of (systematic) reviews on the topic will be examined (i.e. snowballing). Additionally, reversed snowballing is performed by identifying relevant documents that cited the included studies, using the Google Scholar search engine.

iii. Search string

Three domains were included in the search: ‘bacterial infections’, ‘high-throughput sequencing’, and ‘public health’. Each domain has several search terms. The use of wildcards (*) ensured that articles containing any variation of each the search terms were identified. Free text search and MeSH term search were combined. The search was pre-tested to determine the most effective balance of sensitivity and specificity in the identification of potentially relevant citations. The ability of the electronic search to capture all relevant primary research was verified by hand-searching reference lists from other reviews on the topic.

**Domain**	**Search terms**
Bacterial infections	("Bacterial Infections"[Mesh] OR "Microbiology"[Mesh] OR "Genome, Bacterial"[MeSH] OR "Foodborne Diseases"[Mesh] OR "foodborne"[TIAB] OR "microbiology"[TIAB] OR "bacteria*"[TIAB])<br>*1282133 hits*
High-throughput sequencing	("High-Throughput Nucleotide Sequencing"[Mesh] OR "Metagenomics"[Mesh] OR "Genotyping Techniques/methods"[MAJR] OR "whole genome sequencing"[TIAB] OR "next generation sequencing"[TIAB] OR "NGS"[TIAB] OR

	"WGS"[TIAB] OR "genomics"[TIAB] OR "metagenomics"[TIAB])<br>*82159 hits*
Public health	("Public Health Practice"[Mesh] OR "Disease Outbreaks"[Mesh] OR "Epidemiologic Methods"[Mesh] OR "surveillance"[TIAB] OR "outbreak*"[TIAB] OR "epidemiology"[TIAB] OR "monitor*" [TIAB])<br>*6041033 hits*

<u>Search string</u>

("Bacterial Infections"[Mesh] OR "Microbiology"[Mesh] OR "Genome, Bacterial"[MeSH] OR "Foodborne Diseases"[Mesh] OR "foodborne"[TIAB] OR "microbiology"[TIAB] OR "bacteria*"[TIAB]) AND ("High-Throughput Nucleotide Sequencing"[Mesh] OR "Metagenomics"[Mesh] OR "Genotyping Techniques/methods"[MAJR] OR "whole genome sequencing"[TIAB] OR "next generation sequencing"[TIAB] OR "NGS"[TIAB] OR "WGS"[TIAB] OR "genomics"[TIAB] OR "metagenomics"[TIAB]) AND ("Public Health Practice"[Mesh] OR "Disease Outbreaks"[Mesh] OR "Epidemiologic Methods"[Mesh] OR "surveillance"[TIAB] OR "outbreak*"[TIAB] OR "epidemiology"[TIAB] OR "monitor*" [TIAB])

<u>Number of hits</u>

- 2752 hits (PubMed search – 24/03/2018)
- 1536 hits after exclusion of articles before 1/1/2015 (PubMed search – 24/03/2018)
- 142 hits (updated PubMed search – 4/9/2018, with date range selected: ‘1/3/2018 - …’)

SELECTION OF STUDIES

All references identified by the compound search will be imported into an Excel sheet. Duplicates will be removed. A first screening phase based on titles and abstracts will be conducted by NVG and irrelevant studies will be excluded, i.e. excluding the papers that fulfill at least one of the exclusion criteria. Full texts of all potentially relevant studies will be obtained and imported into Mendeley and inclusion criteria will be applied using a standardized eligibility form. This second screening stage based on the full text will be conducted in duplicate by two independent reviewers (NVG, TD). Final agreement on study inclusion will be determined through consensus between the two reviewers. In the case no consensus can be reached, a third reviewer (NB) will help resolve any disagreement. Reference lists of all studies identified by the above methods and bibliographies of reviews and editorials will be examined and additional references satisfying original eligibility criteria will be identified (snowballing and reverse snowballing).

DATA EXTRACTION

Data extraction will be performed using a standardized extraction form (Excel table) by NVG. Charting the results will provide a logical and descriptive summary. This process will be iterative in nature whereby the charting table is continually updated. The following data will be extracted:

Characteristic	Categories
Title	
Author	
Year of publication	
Pathogen	
Country	
Setting	Community, hospital, nursing home, school, etc.
Material sequenced (sample size)	Clinical isolates
	Food isolates
	Animal isolates
	Environmental isolates
Sampling fraction of the NGS analyses	All available samples
	Subset of available samples (complementary)
Public health objective	Outbreak investigation
	Control-oriented surveillance
	Strategy-oriented surveillance
Study aim(s)	Source tracing
	Contact tracing
	Understand transmission dynamics (identify transmission networks/clusters)
	Inform outbreak management: feedback on key phenotypic attributes
	Early outbreak detection
	Overview of circulating strains to identify the emergence of new threats: detection of novel strains/antimicrobial resistance/cross-border epidemic strains/zoonotic events/enhanced virulence
	Impact assessment of prevention and control programs: vaccination/antibiotic stewardship
	Identification of risk factors and risk groups
Time orientation of NGS analyses	Retrospective
	'Near' real-time (within the time period of the outbreak investigation)
	Prospective
Level of implementation of the NGS analyses	Proof-of-concept
	Used for a specific public health problem
	Implemented into routine public health practices: nation-wide prospective genotyping (surveillance)/routine tool during outbreak investigation
Key findings	

PRESENTATION OF THE RESULTS

The results of the search strategy will be presented in a PRISMA flow diagram indicating the number of articles found via each search method, the number of duplicates removed and the number of studies excluded and included.

A great heterogeneity of settings, applications, pathogens, and study measurements is expected. To illustrate and summarize the main findings, results will be presented in tabular form (as per data extraction proforma), with an accompanying narrative summary describing how the results relate to the review objective and question.

The analysis (collating and summarizing) will include:

- Descriptive numerical summary (classification): describing the characteristics of the studies, types of study design, year of publications, study aims, pathogens, countries, etc.
- Thematic analysis: narrative summary of applications, added value and challenges.

QUALITY ASSESSMENT

The review will not assess the quality of the evidence identified from the literature, rather provide an overview of the existing evidence, regardless of quality. Following typical scoping review methods, methodological quality of the included articles will not be assessed systematically, however only peer-reviewed articles will be included.

REFERENCES

1. Kan B, Zhou H, Du P, Zhang W, Lu X, Qin T, et al. Transforming bacterial disease surveillance and investigation using whole-genome sequence to probe the trace. Front Med. 2018;12(1):23–33.
2. Pham MT, Rajić A, Greig JD, Sargeant JM, Papadopoulos A, Mcewen SA. A scoping review of scoping reviews: Advancing the approach and enhancing the consistency. Res Synth Methods. 2014;
3. Arksey H, O'Malley L. Scoping studies: Towards a methodological framework. Int J Soc Res Methodol Theory Pract. 2005;8(1):19–32.
4. Lucyk K, McLaren L. Taking stock of the social determinants of health: A scoping review. PLoS One [Internet]. 2017;12(5). Available from: https://doi.org/10.1371/journal.pone.0177306
5. Tao D, Evashwick CJ, Grivna M, Harrison R. Educating the Public Health Workforce: A Scoping Review. Front Public Heal. 2018;6(27).
