# Supplementary material for: Status and potential of bacterial genomics for public health practice: a scoping review
Source: Implement Sci. 2019 Aug 13;14:79. doi: 10.1186/s13012-019-0930-2 (PMC6692930; doi:10.1186/s13012-019-0930-2)
Supplement: Supplementary file 3 — Inclusion and exclusion criteria organized according to the Population-Concept-Context (PCC) principle, as outlined in the Joanna Briggs’ Manual for Scoping Reviews. (PDF 499 kb) [file 13012_2019_930_MOESM3_ESM.pdf]

Inclusion	Exclusion
Language: English full text<br>Timeframe: studies published between 1/1/2015 and 4/9/2018<br>All geographical regions<br>Studies with primary NGS data available or describing the implementation of NGS	No English full text available<br>Studies published before 1/1/2015<br>/<br>Conference abstracts or literature reviews
Population	
Studies must include at least two individuals with a bacterial infection.	Non-human studies, i.e. studies only including environmental or animal samples.
Concept	
Studies must apply NGS on the bacterial isolates (pathogen genomics), more specifically studies with a strong focus on the use of NGS (as a primary typing tool or being a determining factor for the outcome of the study)	No use of NGS (e.g. microarray studies).<br>Use of NGS for a small, insignificant subset of samples, i.e. studies where NGS does not play a major role and/or where there is not focus on the use/application/added value/challenges of NGS.<br>Not applying NGS on bacterial isolates (e.g. studies only sequencing host genomes, studies only sequencing pathogens other than bacteria).
Context	
To be considered genomic epidemiology, study aims should include at least one of the following:<br>- Outbreak investigation (source tracing, interrupt transmission, feedback on key phenotypic attributes)<br>- Control-oriented surveillance (early outbreak detection, identify the emergence of new threats, understand transmission dynamics)<br>- Strategy-oriented surveillance (overview of circulating strains, understand transmission dynamics, evaluation of control programs, identification of risk factors)	Studies that do not focus on outbreak investigations or surveillance and control (i.e. not for public health practice), for example:<br>- Studies only focusing on technical aspects of the sequence technologies (e.g. benchmarking of sequencing methods, comparison of sequencing methods, description of sequencing methods, etc.)<br>- Studies only/mainly focusing on bioinformatics aspects (e.g. developing new typing schemes)<br>- Studies using NGS for research purposes (e.g. underlying biological mechanisms, cellular processes, evolutionary aspects, etc.)<br>- Studies using NGS to identify biological markers that can be used for future routine public health practices
Studies must describe the application of NGS at the population level (i.e. from a public health perspective)	Studies not focusing on the population perspective:<br>- Studies using NGS solely for individual patient care (e.g. diagnostic studies - studies examining whole genome sequencing for prediction of phenotypic drug resistance or studies that exclusively test new diagnostic approaches without meeting the above inclusion criteria)<br>- Studies including less than two clinical samples from patients, apart from environmental or animal samples.<br>- Studies describing individual patients/cases (i.e. case studies)
Studies using NGS within a real-life public health setting producing an output that can be directly translated into actionable results for public health (including proof-of-concept studies).	Studies using NGS in an experimental setting (e.g. using a small convenience sample of historic isolates, only suggesting the use of NGS in a routine public health setting but not yet applying it, etc.)
