# Supplementary material for: Status and potential of bacterial genomics for public health practice: a scoping review
Source: Implement Sci. 2019 Aug 13;14:79. doi: 10.1186/s13012-019-0930-2 (PMC6692930; doi:10.1186/s13012-019-0930-2)
Supplement: Supplementary file 4 — Decision tree used during screening to distinguish between research, individual patient care, and public health practice. (PDF 275 kb) [file 13012_2019_930_MOESM4_ESM.pdf]

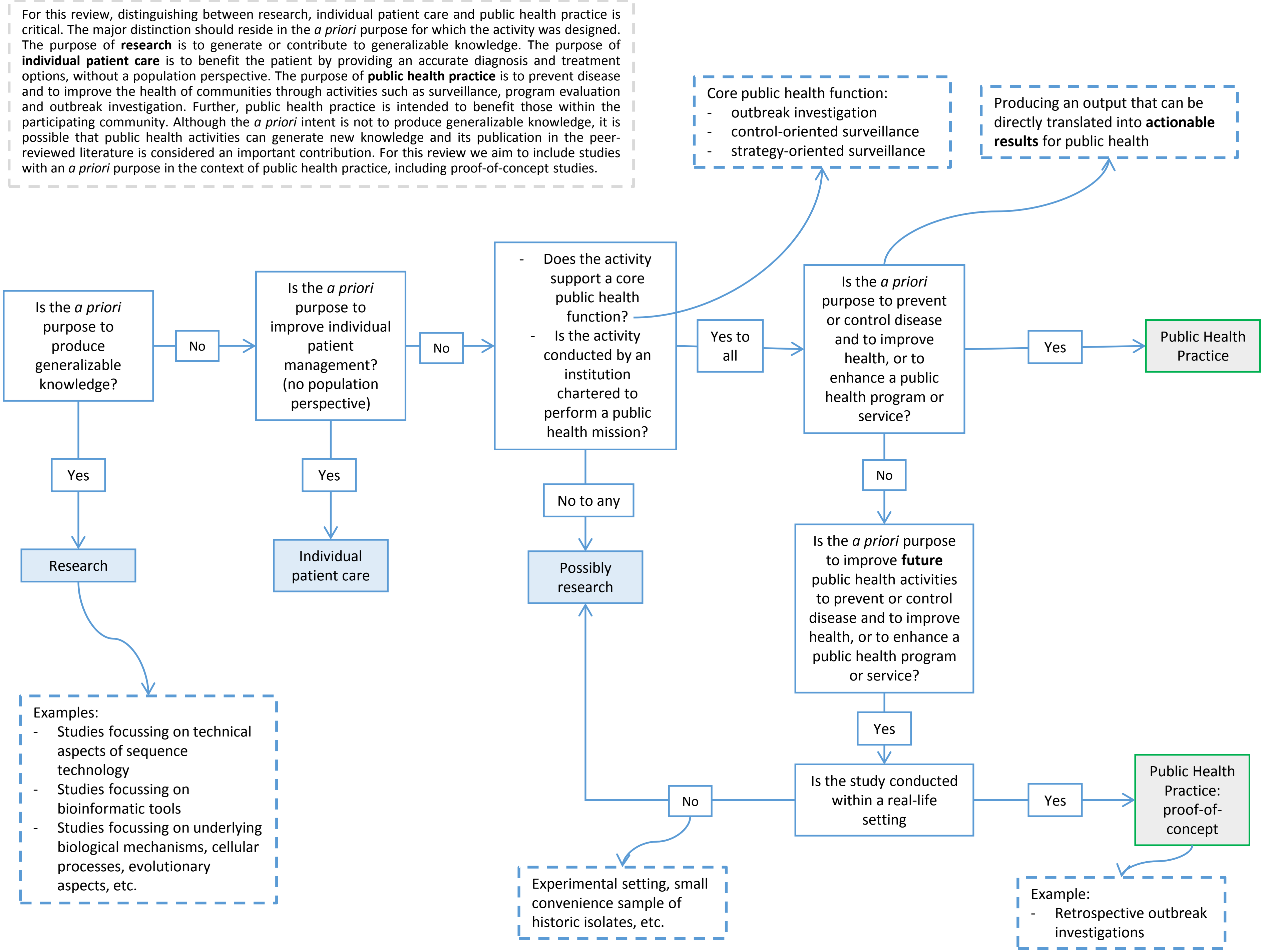
For this review, distinguishing between research, individual patient care and public health practice is critical. The major distinction should reside in the *a priori* purpose for which the activity was designed. The purpose of **research** is to generate or contribute to generalizable knowledge. The purpose of **individual patient care** is to benefit the patient by providing an accurate diagnosis and treatment options, without a population perspective. The purpose of **public health practice** is to prevent disease and to improve the health of communities through activities such as surveillance, program evaluation and outbreak investigation. Further, public health practice is intended to benefit those within the participating community. Although the *a priori* intent is not to produce generalizable knowledge, it is possible that public health activities can generate new knowledge and its publication in the peer-reviewed literature is considered an important contribution. For this review we aim to include studies with an *a priori* purpose in the context of public health practice, including proof-of-concept studies.
Core public health function:
- outbreak investigation
- control-oriented surveillance
- strategy-oriented surveillance
Producing an output that can be directly translated into **actionable results** for public health
Is the *a priori* purpose to produce generalizable knowledge?
No
Is the *a priori* purpose to improve individual patient management? (no population perspective)
No
- Does the activity support a core public health function?
- Is the activity conducted by an institution chartered to perform a public health mission?
Yes to all
Is the *a priori* purpose to prevent or control disease and to improve health, or to enhance a public health program or service?
Yes
Public Health Practice
Yes
Research
Yes
Individual patient care
No to any
Possibly research
No
Is the *a priori* purpose to improve **future** public health activities to prevent or control disease and to improve health, or to enhance a public health program or service?
Yes
Is the study conducted within a real-life setting
Yes
Public Health Practice: proof-of-concept
No
Examples:
- Studies focussing on technical aspects of sequence technology
- Studies focussing on bioinformatic tools
- Studies focussing on underlying biological mechanisms, cellular processes, evolutionary aspects, etc.
Experimental setting, small convenience sample of historic isolates, etc.
Example:
- Retrospective outbreak investigations
