# Supplementary material for: Status and potential of bacterial genomics for public health practice: a scoping review
Source: Implement Sci. 2019 Aug 13;14:79. doi: 10.1186/s13012-019-0930-2 (PMC6692930; doi:10.1186/s13012-019-0930-2)
Supplement: Supplementary file 5 — PubMed search string. (PDF 176 kb) [file 13012_2019_930_MOESM5_ESM.pdf]

Domain	Search terms
Bacterial infections	("Bacterial Infections"[Mesh] OR "Microbiology"[Mesh] OR "Genome, Bacterial"[MeSH] OR "Foodborne Diseases"[Mesh] OR "foodborne"[TIAB] OR "microbiology"[TIAB] OR "bacteria*"[TIAB]<br><br>AND
Next generation sequencing	"High-Throughput Nucleotide Sequencing"[Mesh] OR "Metagenomics"[Mesh] OR "Genotyping Techniques/methods"[MAJR] OR "whole genome sequencing"[TIAB] OR "next generation sequencing"[TIAB] OR "NGS"[TIAB] OR "WGS"[TIAB] OR "genomics"[TIAB] OR "metagenomics"[TIAB]<br><br>AND
Public health	"Public Health Practice"[Mesh] OR "Disease Outbreaks"[Mesh] OR "Epidemiologic Methods"[Mesh] OR "surveillance"[TIAB] OR "outbreak*"[TIAB] OR "epidemiology"[TIAB] OR "monitor*" [TIAB]

PubMed search string used on 24/03/2018 and on 04/09/2019 (for the updated search)

("Bacterial Infections"[Mesh] OR "Microbiology"[Mesh] OR "Genome, Bacterial"[MeSH] OR "Foodborne Diseases"[Mesh] OR "foodborne"[TIAB] OR "microbiology"[TIAB] OR "bacteria*"[TIAB]) AND ("High-Throughput Nucleotide Sequencing"[Mesh] OR "Metagenomics"[Mesh] OR "Genotyping Techniques/methods"[MAJR] OR "whole genome sequencing"[TIAB] OR "next generation sequencing"[TIAB] OR "NGS"[TIAB] OR "WGS"[TIAB] OR "genomics"[TIAB] OR "metagenomics"[TIAB]) AND ("Public Health Practice"[Mesh] OR "Disease Outbreaks"[Mesh] OR "Epidemiologic Methods"[Mesh] OR "surveillance"[TIAB] OR "outbreak*"[TIAB] OR "epidemiology"[TIAB] OR "monitor*" [TIAB])
