# Supplementary material for: Status and potential of bacterial genomics for public health practice: a scoping review
Source: Implement Sci. 2019 Aug 13;14:79. doi: 10.1186/s13012-019-0930-2 (PMC6692930; doi:10.1186/s13012-019-0930-2)
Supplement: Supplementary file 6 — Extraction form listing the characteristics that are extracted from the included studies. (PDF 179 kb) [file 13012_2019_930_MOESM6_ESM.pdf]

Characteristic	Categories	Remarks/explanation
Title		
Author		
Year of publication		
Pathogen		
Country		Country of residence of the patients infected with the bacteria
Setting	Community, hospital, nursing home, school, etc.	Community vs. Institutional
Material sequenced (sample size)	Clinical isolates	
	Food isolates	
	Animal isolates	
	Environmental isolates	
Sampling fraction of the NGS analyses	All available samples	Which proportion (all or subset) of the samples that were collected/available for this particular study (surveillance/outbreak investigation) were sequenced?
	Subset of available samples (complementary)	
Public health objective	Outbreak investigation	As a reaction to a sudden increase in the number of cases or to an unusual event. The main objective is to investigate the possible source(s) of infection and/or routes of transmission, and to implement effective and appropriate control measures to stop its further spread.
	Control-oriented surveillance	The purpose is to identify each occurrence of a particular disease, hazard, or other health-related event that requires a specific response, and to support the delivery of an effective intervention.
	Strategy-oriented surveillance	The aim is to monitor long-term changes in epidemiology over larger geographic and population scales and to provide information to support prevention strategies to reduce population risk.
Study aim(s)	Source tracing	
	Contact tracing	
	Understand transmission dynamics (identify transmission networks/clusters)	
	Inform outbreak management: feedback on key phenotypic attributes	
	Early outbreak detection	
	Overview of circulating strains to identify the emergence of new threats: detection of novel strains/antimicrobial resistance/cross-border epidemic strains/zoonotic events/enhanced virulence	
	Impact assessment of prevention and control programs: vaccination/antibiotic stewardship	
	Identification of risk factors and risk groups	
Time orientation of NGS analyses	Retrospective	
	'Near' real-time (within the time period of the outbreak investigation)	
	Prospective	
Level of implementation of the NGS analyses	Proof-of-concept	
	Used for a specific public health problem	
	Implemented into routine public health practices: nation-wide prospective genotyping (surveillance)/routine tool during outbreak investigation	
Key findings		Reported key findings concerning the use of pathogen genomics for public health practice in terms of its added value and challenges.
